# Cancer organoid-based diagnosis reactivity prediction (CODRP) index-based anticancer drug sensitivity test in ALK-rearrangement positive non-small cell lung cancer (NSCLC)

**DOI:** 10.1186/s13046-023-02899-4

**Published:** 2023-11-22

**Authors:** Sang-Yun Lee, Hyeong Jun Cho, Jimin Choi, Bosung Ku, Seok Whan Moon, Mi Hyoung Moon, Kyung Soo Kim, Kwanyong Hyun, Tae-Jung Kim, Yeoun Eun Sung, Yongki Hwang, Eunyoung Lee, Dong Hyuck Ahn, Joon Young Choi, Jeong Uk Lim, Chan Kwon Park, Sung Won Kim, Seung Joon Kim, In-Seong Koo, Woo Seok Jung, Sang-Hyun Lee, Chang Dong Yeo, Dong Woo Lee

**Affiliations:** 1 Department of Biomedical Engineering, Gachon University, Seongnam, 13120 Republic of Korea; 2 Central R & D Center, Medical & Bio Decision (MBD) Co., Ltd, Suwon, 16229 Republic of Korea; 3 Division of Pulmonology, Department of Internal Medicine, Seoul St. Mary’s Hospital, College of Medicine, The Catholic University of Korea, Seoul, Republic of Korea; 4 Department of Thoracic and Cardiovascular Surgery, College of Medicine, The Catholic University of Korea, Seoul, 06591 Korea; 5 Department of Hospital Pathology, College of Medicine, The Catholic University of Korea, Seoul, 06591 Korea; 6 Division of Pulmonary and Critical Care Medicine, Department of Internal Medicine, Incheon St. Mary’s Hospital, College of Medicine, The Catholic University of Korea, Seoul, Republic of Korea; 7 Division of Pulmonary, Critical Care and Allergy, Department of Internal Medicine, Yeouido St. Mary’s Hospital, College of Medicine, The Catholic University of Korea, Seoul, Republic of Korea; 8 Department of Otorhinolaryngology-Head and Neck Surgery, College of Medicine, The Catholic University of Korea, Seoul, Republic of Korea; 9 Department of Biomedicine & Health Sciences, College of Medicine, The Catholic University of Korea, Seoul, Republic of Korea; 10 Postech-Catholic Biomedical Engineering Institute, College of Medicine, The Catholic University of Korea, Songeui Multiplex Hall, Seoul, Republic of Korea; 11 Division of Pulmonary and Critical Care Medicine, Department of Internal Medicine, Eunpyeong St. Mary’s Hospital, College of Medicine, The Catholic University of Korea, Seoul, Republic of Korea

**Keywords:** Non-small cell Lung cancer (NSCLC), High-throughput screening (HTS), Cancer Organoid-based diagnosis reactivity prediction (CODRP) platform, Patient-derived Organoid (PDO), 3D cell culture

## Abstract

**Background:**

Recently, cancer organoid-based drug sensitivity tests have been studied to predict patient responses to anticancer drugs. The area under curve (AUC) or IC_50_ value of the dose-response curve (DRC) is used to differentiate between sensitive and resistant patient‘s groups. This study proposes a multi-parameter analysis method (cancer organoid-based diagnosis reactivity prediction, CODRP) that considers the cancer stage and cancer cell growth rate, which represent the severity of cancer patients, in the sensitivity test.

**Methods:**

On the CODRP platform, patient-derived organoids (PDOs) that recapitulate patients with lung cancer were implemented by applying a mechanical dissociation method capable of high yields and proliferation rates. A disposable nozzle-type cell spotter with efficient high-throughput screening (HTS) has also been developed to dispense a very small number of cells due to limited patient cells. A drug sensitivity test was performed using PDO from the patient tissue and the primary cancer characteristics of PDOs were confirmed by pathological comparision with tissue slides.

**Results:**

The conventional index of drug sensitivity is the AUC of the DRC. In this study, the CODRP index for drug sensitivity test was proposed through multi-parameter analyses considering cancer cell proliferation rate, the cancer diagnosis stage, and AUC values. We tested PDOs from eight patients with lung cancer to verify the CODRP index. According to the anaplastic lymphoma kinase (ALK) rearrangement status, the conventional AUC index for the three ALK-targeted drugs (crizotinib, alectinib, and brigatinib) did not classify into sensitive and resistant groups. The proposed CODRP index-based drug sensitivity test classified ALK-targeted drug responses according to ALK rearrangement status and was verified to be consistent with the clinical drug treatment response.

**Conclusions:**

Therefore, the PDO-based HTS and CODRP index drug sensitivity tests described in this paper may be useful for predicting and analyzing promising anticancer drug efficacy for patients with lung cancer and can be applied to a precision medicine platform.

**Supplementary Information:**

The online version contains supplementary material available at 10.1186/s13046-023-02899-4.

## Background

Anaplastic lymphoma kinase (ALK) rearrangement-positive lung cancer is a subtype of lung cancer and accounts for approximately 5% of the total non-small cell lung cancers (NSCLC) driven by a genetic alteration of the ALK gene [1–5]. A fusion gene showing the inversion of ALK and echinoderm microtubule-associated protein-like 4 (EML4), which produces a constitutively activated ALK protein, triggers abnormal signaling pathways that promote uncontrolled cell growth and survival. As such, this fusion gene has been used as a therapeutic target since 2007 [6, 7]. Treatment options for ALK rearrangement-positive NSCLC include targeted therapy with ALK inhibitors [8, 9], such as crizotinib, ceritinib, alectinib, brigatinib, and lorlatinib. These drugs work by inhibiting the activity of abnormal ALK proteins, leading to tumor shrinkage and improved survival. Despite the development and increased availability of new therapeutic drugs for lung cancer, ALK-targeted drugs show a varied spectrum of drug efficacy and resistance to various mutations, thus leaving lung cancer the leading cause of cancer-related mortality worldwide [10]. Owing to these variations, clinicians require considerable time to determine the treatment direction [11]. In other words, clinical unmet needs remain a hindrance to quickly obtaining anticancer drug sensitivity results through cancer patient-derived samples.

Accurate prediction of the drug efficacy of therapeutic candidates is one of the most important processes in precision medicine [12, 13]. Although precision medicine based on genome analyses has been attempted previously, limitations exist in predicting the association between genetic variations and targeted drug efficacy due to tumor heterogeneity [14–16]. In addition, in vivo cancer models, such as patient-derived xenografts (PDXs), have limitations because of their low success rate, high cost, and time consumption [17, 18]. Research on an in vitro tumor model-based precision medicine platform using patient-derived organoids (PDOs) has recently been conducted to overcome these limitations. PDOs were applied in high-throughput screening (HTS) models better to mimic solid tumors’ physiological properties in patients with cancer and rapidly predict patient-specific drug responses [19, 20]. As such, PDO is being used in functional-precision oncology research to implement precision medicine. It is also considered an important diagnostic tool that can assist clinical decision-making and accelerate the development of therapeutic strategies [21–23]. However, the current PDO-based drug sensitivity prediction test is inadequate due to the limited number of samples that can be obtained from patients. Moreover, since the conventional HTS analyses [24] required sufficient cells, additional cell expansion culture is required. In addition, the algorithm for analyzing drug efficacy is only dependent on the Area Under Curve (AUC) of the dose-response curve (DRC) [25] which did not regarding individual patient’s conditions such as cancer stage and cell growth.

This study addresses the aforementioned problems by developing a disposable nozzle-type cell spotter and culturing PDOs in a 384-pillar/well plate for minimalized cell number in PDO-based drug screening platform. Furthermore, the study proposes a cancer organoid-based diagnosis reactivity prediction (CODRP) analysis algorithm to improve drug sensitivity prediction accuracy. CODRP algorithm used multi-parameters such as individual patient’s cancer stage and organoid growth as well as the AUC of the DRC. The disposable nozzle-type cell spotter was shown to improve the repetitive equipment washing process and cross-contamination of patient samples by automatically dispensing a small number of patient-derived lung cancer cells (PDCs) on the surface of a 384-pillar plate [26–28]. To compare the performance of the conventional AUC-based drug sensitivity test and the proposed CODRP drug sensitivity test algorithm, PDOs from ALK-positive and negative lung cancers were tested for three ALK-targeted drugs (crizotinib, alectinib, and brigatinib). We then compared the drug sensitivity results with the clinical outcomes of patients who were treated with the same drugs. The proposed CODRP algorithm better discriminates ALK-targeted drug responses according to ALK mutations by considering multi-parameters (cancer stage, cell growth rate). CODPR drug sensitivity test results also show good agreement with clinical patient responses to ALK-targeted drugs. Based on the results obtained, we suggest that the PDO HTS-based CODRP sensitivity test algorithm is more suitable for the evaluation of targeted drugs in patients with lung cancer than the conventional HTS drug sensitivity test model.

## Methods

### Development of disposable nozzle-type cell spotter

**Fig. 1:**
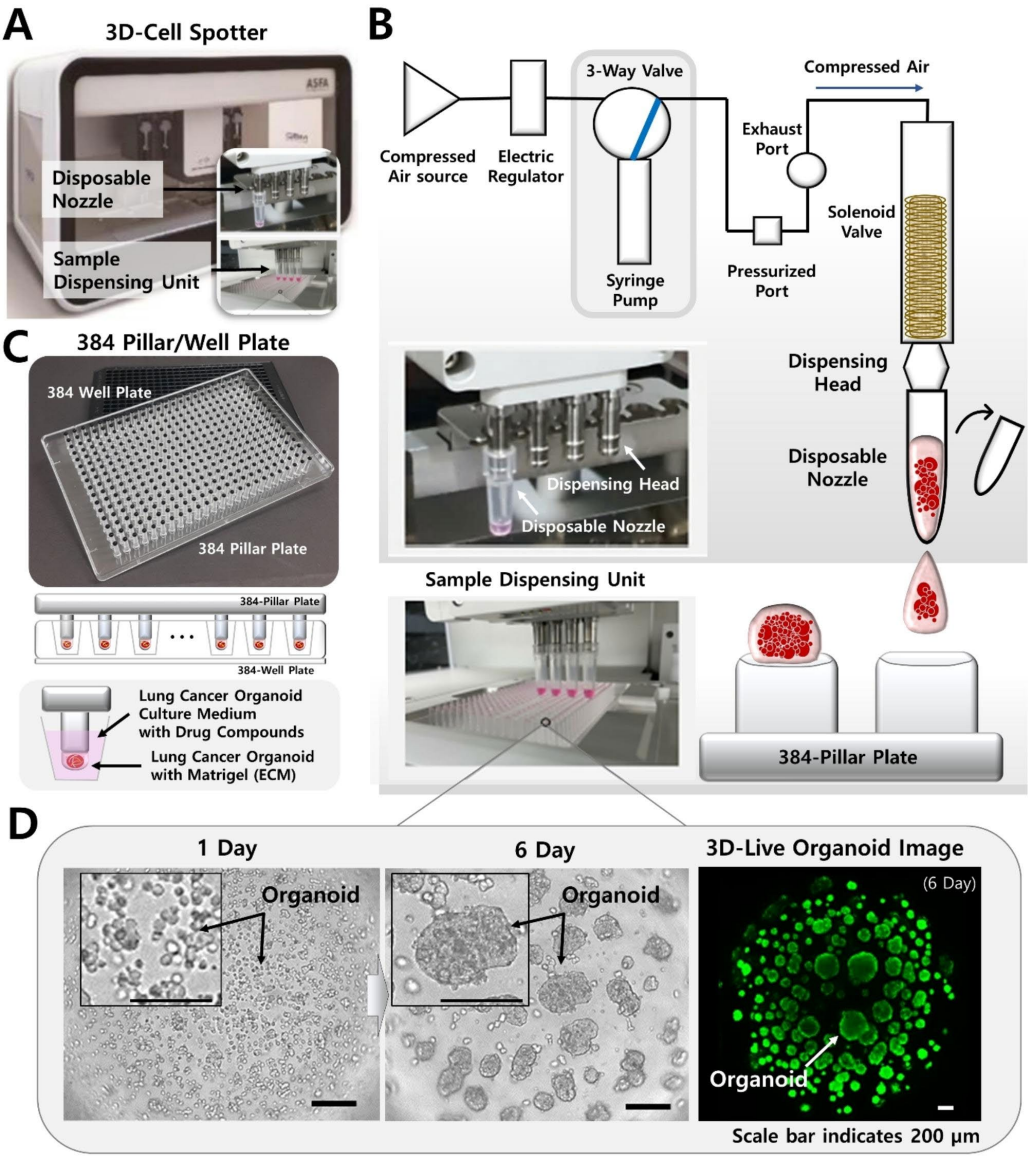
A Schematic view of high-throughput screening (HTS) platform using patient-derived lung cancer organoids (PDOs). (**A**) Photographs of the proposed cell spotter (ASFA™ Spotter DN) with disposable nozzle and sample dispensing unit. (**B**) Schematic diagram of module parts for dispensing of ASFA™ Spotter DN. (**C**) 384-pillar/well plate photograph and 3D cell culture illustration. The lung cancer PDO is mixed with an ECM and is 3D cultured and drug screen on the surface of a 384-pillar plate. (**D**) Representative bright field (BF) and confocal images of PDOs from day 1 to 6. The lung cancer PDO grows by forming colonies

The cancer cells were separated into single cells by enzymatic treatment and prepared to contain approximately 5000 cells and 80% Matrigel (80 v/v) per 1 µL volume. The conventional cell spotter dispensing cells uses stainless nozzle and a solenoid valve (The Lee Company, USA) to dispense 1 µL droplets of a cancer cell-hydrogel mixture onto the target plate. This process, however, requires repeated washing steps every time the sample and drug are exchanged. To overcome these disadvantages, a disposable nozzle-type cell potter (ASFATM Spotter DN, Medical & Bio Decision, South Korea) was developed with a level of precision for biological experiments, allowing high-throughput automatic sample dispensing in the unit (Fig. 1A). The cell spotter dispenses liquid samples using a force that pushes the syringe pump (Figure S1). It consists of an electric regulator, syringe pump, dispensing head, and disposable nozzles that quantitatively dispense a liquid sample by controlling the pressure generated from the compressed air source using an electric regulator (Fig. 1B and Figure S1B). On loading the prepared PDC-Matrigel mixtures into the source plate well, the conventional cell spotter aspirates the sample using negative force and dispenses it on the surface of the target 384-pillar plate. This process occurs in a high-throughput manner (Fig. 1C). The viscous PDC-Matrigel mixtures were uniformly dispensed onto the target plate due to the electric regulator’s fine pressure control and the rapid sample aspiration process using the syringe pump. The PDCs were uniformly dispensed in a 384-well plate containing fresh culture medium and drug compounds to form PDC colonies that were successfully cultured as PDOs (Fig. 1D).

### Preparation of the 384-pillar/well plates

The 384-pillar plate is made of poly (styrene-co-maleic anhydride) (PS-MA) and contains 384-pillars (each with a 2 mm pillar diameter and 4.5 mm pillar-to-pillar distance), manufactured by plastic injection molding (Fig. 1C). Plastic molding was performed using an injection molder (Sodic Plustech Inc., IL, USA). PS-MA, a widely used biocompatible plastic, is used to prepare 384-pillar/well plates, making them the most robust and flexible material suitable for mammalian cell culture, enzymatic reactions, viral infections, and compound screening. To prevent contamination and polymerization of Matrigel, the surface of the 384-pillar plate was plasma-treated for 10 s (80 W power, 5 × 10^− 4^ Torr using air) and coated with diluted laminin solution (L2020-1 mg, Sigma, St. Louis, MO, USA) in PBS. To prepare a laminin coating solution, 10 mL of phosphate-buffered saline (PBS) was mixed with a 1/100 ratio of pure laminin solution (1 mg/mL). The 384-pillar plate can be combined with a commercial 384-well plate to incubate PDOs for targeted anticancer drug efficacy analyses.

### Calculation of drug response in PDO-based HTS (AUC index)

**Fig. 2:**
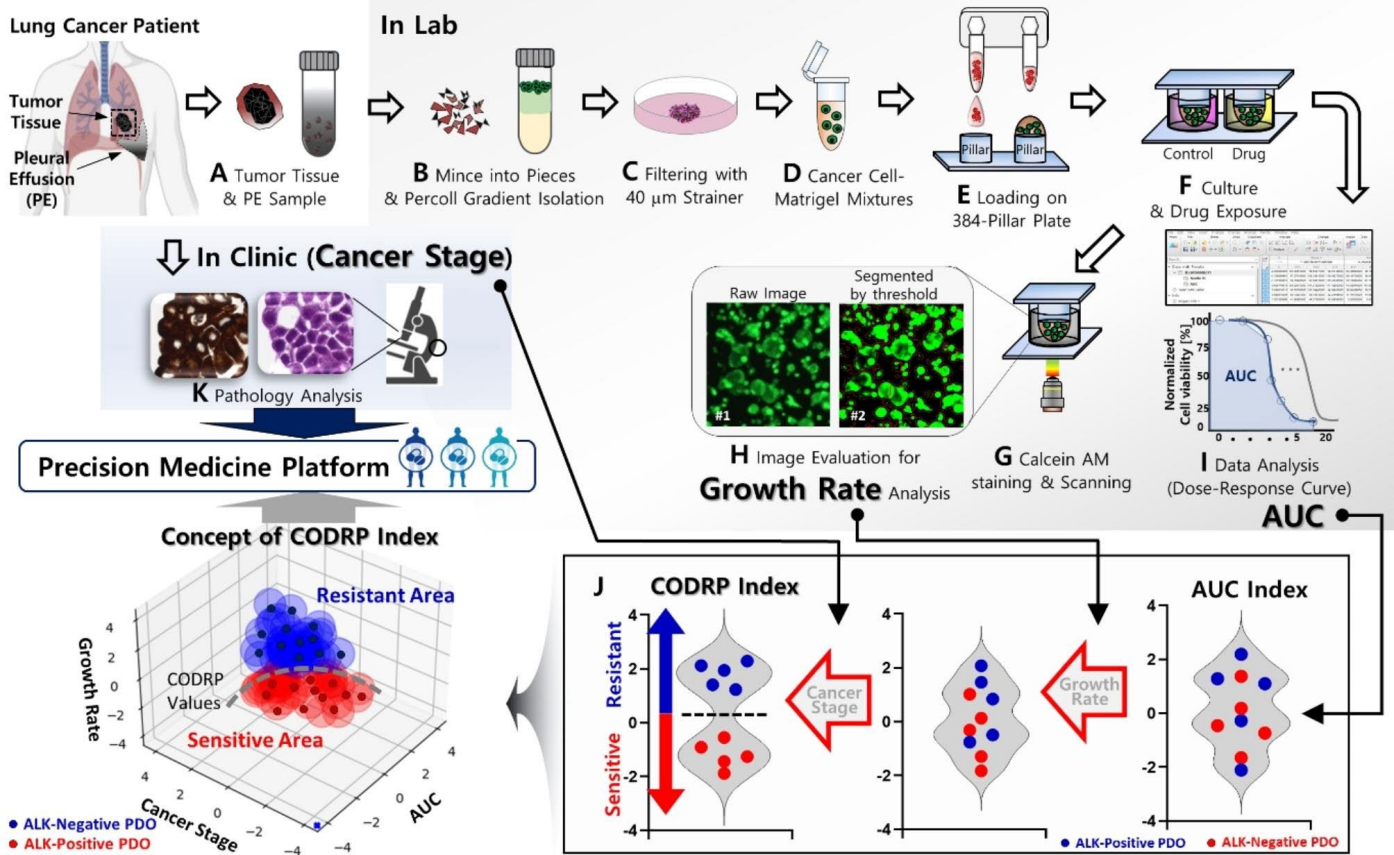
Experimental procedures of HTS using lung cancer PDOs (**A**) Tumor tissue and malignant pleural effusion (MPE) samples derived from patients with lung cancer. (**B**) Preparation step for isolating cancer cells from patient-derived lung cancer tumor tissues and MPEs. (**C**) The pretreated patient-derived lung cancer samples filtered using a 40 μm strainer. (**D**) Patient-derived lung cancer cells mixed with the extracellular matrix (ECM) of hydrogel components. (**E**) Cancer cell and Matrigel mixtures dispensed on the surface of 384-pillar plate by ASFA™ Spotter DN. (**F**) The drug exposed by combining the 384-pillar plate, in which the cells have been dispensed, with the 384-well plates containing the fresh culture medium and drugs. (**G**) 3D live cell staining by replacing the 384-well plate with one filled with live cell-staining dye; images were taken after live cell staining. (**H**) Quantifying the percentage of live cells by evaluating scanned images and calculating the cell growth rate. (**I**) Cell viability measurement through CellTiter-Glo reagent treatment and luminescence value readout; drug sensitivity analysis based on AUC index from dose-response curve (DRC). (**J**) CODRP index predicted drug efficacy by considering the cell growth rate and cancer diagnosed stage in the AUC index. (**K**) Immunohistochemistry (IHC) analysis using patient-derived lung cancer tissues and cultured lung cancer organoid samples

Surgical tumor tissue and malignant pleural effusion (MPE) samples were obtained from patients diagnosed with lung cancer (Fig. 2A). Through the patient-derived pretreatment process described below, the tumor tissue was mechanically divided, and MPE samples were separated from patient-derived cancer cells by the Percoll concentration (Fig. 2B). The isolated PDCs were filtered with a 40 μm strainer (Fig. 2C) and then prepared at a concentration of approximately 5000 cells and 80% Matrigel (80 v/v) per 1.5 µL volume (Fig. 2D). The prepared PDC-Matrigel mixtures were automatically dispensed onto 384-pillar plates using an ASFA™ Spotter DN. The ASFA™ Spotter DN uses a disposable nozzle to dispense 1.5 µL droplets of the PDC–Matrigel mixtures on the 384-pillar plate surface (Fig. 2E). The PDC-Matrigel mixtures were then sandwiched (or “stamped”) between the 384-pillar plate they were dispensed on and the 384-well plate for PDO culture and drug exposure (Fig. 2F). The PDC-dispensed 384-pillar plate was combined with the 384-well plate containing the fresh culture medium and pre-cultured for three days in a 5% CO_2_-humidified incubator to form PDOs. ALK inhibitors (crizotinib, alectinib, and brigatinib), targeting major oncogenic signaling molecules, were purchased from AdooQ Bioscience (Irvine, CA, USA) and dissolved in a stock solution of 100 mM dimethyl sulfoxide (DMSO). These drugs were dispensed using the non-contact drug fast-dispensing mode of ASFA™ Spotter DN. The 384-well plate was divided into 12 regions. Each region comprised a 3 × 7 well array corresponding to the three ALK-targeted drugs in a 3-fold and 7-point serial dilution series from 50 µM (including one DMSO control) and individual drugs were tested under three technical replicate conditions. The 3D-cultured PDOs were incubated at 37 °C in a 5% CO_2_ humidified incubator to be exposed to the three ALK-targeted drugs for three days. After incubation, the 384-pillar plate, in which lung cancer cells were cultured, was combined with a new 384-well plate containing a live-cell staining solution for specific staining of living cells after the drug treatment. The staining solution was prepared by adding 1 µL calcein AM to 7 mL Roswell Park Memorial Institute (RPMI) medium. Cells were incubated with the staining solution for 1 h at 37 °C in a 5% CO_2_ humidified atmosphere. Live cell images with green fluorescence intensities (excitation/emission, 494/517 nm from lasers) were scanned using an optical scanner (ASFA™ Scanner HE; Medical & Bio Decision, South Korea) (Fig. 2G). Scanned images were evaluated using image analysis software (ASFA™ Ez SW; Medical & Bio Decision, South Korea), and the growth rate of PDO was calculated from days 1 to 3. (Fig. 2H). After imaging, cell viability was determined using an adenosine triphosphate (ATP) monitoring system based on firefly luciferase (CellTiter-Glo® Cell Viability Assay, Promega, Madison, WI), according to the manufacturer’s protocol. The ATP assay mixture was prepared by adding 20 µL of CellTiter-Glo reagent to 20 µL of the medium per well. Cells were incubated at room temperature for 30 min to stabilize the luminescence signal, and luminescence was recorded using a SpectraMax iD3 Reader (Molecular Devices LLC, San Jose, CA, USA). We performed a specific and accurate drug response analysis using a DRC according to the concentration gradient (GraphPad Prism 9; GraphPad Software, CA, USA). The graph confirmed the response to the three ALK-targeted drugs in individual PDOs through quantified AUC indices (Fig. 2I and Table S1). AUC index is convered to the standard score (Z-score) of AUC. Using the mean and standard deviation of the AUCs of the three ALK-targeted drugs in the individual PDOs, the AUC index was calculated as follows:

1 $${AUC Index }_{drug}={Z score of AUC }_{drug}= \frac{{AUC }_{drug }- mean}{SD}$$

### Calculation of CODRP index (multiple linear regression; MLR analysis)

The diagnosis stage was scored and converted to a Z-score for the entire patient population (Table S2). The growth rate of PDO was measured by the area value of live PDO that increased during the 3 days of drug treatment and then converted to a Z-score. In addition, the CODRP index was calculated by comprehensively considering the scored lung cancer stages, the growth rate of individual PDO, and the AUC value through multiple linear regression (MLR) analysis (GraphPad Prism 9; GraphPad Software, CA, USA) and converted to a Z-score.

The CODRP was calculated as follows:

2 $$CODR{P_{drug}} = {\beta _0} + {\beta _1}{X_1} + {\beta _2}{X_2} + {\beta _3}{X_3}$$

*X*_*1*_, *X*_*2*_, and *X*_*3*_ represent independent variables (cancer stage, growth rate, and AUC index of ALK-targeted drugs), and the constant values (*β*_*0*_, *β*_*1*_, *β*_*2*_, and *β*_*3*_) calculated by MLR analysis are shown in Table S7.

The CODRP index was calculated as follows:

3 $${CODRP\, Index }_{drug}={Z\, score\, of \,\text{C}\text{O}\text{D}\text{R}\text{P} }_{drug}= \frac{{CODRP }_{drug }- mean}{SD}$$

The CODRP algorithm is a multi-parameter analysis based on MLR analysis using cancer stage, organoid growth rate, and AUC index of individual lung cancer patients. (Table S1). Therefore, as suggested in the CODRP algorithm conceptual diagram, the optimal CODRP algorithm was proposed based on MLR analysis to be able to classify sensitivity to ALK-targeted drugs according to the ALK-rearrangement positive and negative status of PDO (Fig. 2J). To evaluate the performance of drug sensitivity analysis based on AUC and CODRP indices, sensitivity and specificity were calculated compared with the ALK mutation status of clinical patients (Figs. 5C and 6 C). Sensitivity and specificity were calculated as follows:

4 $${Sensitivity_{drug}} = A/(A + C) \times 100$$

5 $${Specificity_{drug}} = D/(B + D) \times 100$$

(A: Number of ALK positive and prediction sensitive cases, B: Number of ALK negative and prediction sensitive cases, C: Number of ALK positive and prediction resistant cases, D: Number of ALK negative and prediction resistant cases)

### Experimental protocol of percoll-gradient centrifugation for PDC isolation from malignant pleural effusion (MPE)

Percoll stock solution was mixed with Hank’s balanced salt solution (HBSS, with phenol red) at 90% (90 v/v). The Percoll stock solution was diluted with a buffer solution composed of 10% fetal bovine serum (FBS) and 100 µM ethylenediamine tetraacetic acid (EDTA) at concentrations of 40% (40 v/v) and 75% (75 v/v). Upon arrival at the laboratory, MPEs (40–60 mL) were transferred to 50 mL tubes and centrifuged at 1500 rpm for 5 min. The cell pellet was rinsed with RPMI medium and filtered through a 40 μm strainer, the cells remaining on the membrane were collected through a wash process, and impurities were discarded. The cell pellet was resuspended in 75% Percoll solution and transferred to a 15 mL tube containing 75% Percoll solution to a total volume of 5 mL. To prevent mixing of the two solutions, 5 mL of a 40% Percoll solution was gently added to the tube and centrifuged at 2000 rpm for 20 min without interruption. The cells, separated into upper and middle layers owing to different densities, were transferred to individual tubes and washed with RPMI medium. CD3-negative cells in the upper layer were considered PDC and were used for PDO culture.

### Experimental protocol of PDC isolation from patient tissues with lung cancer tumor

After obtaining patient consent, all patient-derived lung cancer samples and clinical data were collected at Seoul St. Mary’s Hospital (The Catholic University). Tumor tissue and MPE were obtained from patients with lung cancer, diagnosed with lung adenocarcinoma, by surgical excision, and transported to the laboratory. Tumor tissues were cut into small pieces using surgical scissors on a 60 mm dish containing a cold PRMI medium. To increase the yield of PDC, we proposed a mechanical dissociation method by replacing the conventional enzymatic dissociation method to separate tumor tissue into single cells (Figure S1). The conventional enzymatic dissociation uses cocktails composed of collagenase, DNase I, and dispase to digest the tissue. The minced tumor tissues were rinsed with cold RPMI medium to remove debris and blood, and then incubated in advanced Dulbecco’s Modified Eagle Medium/Nutrient Mixture F-12 (DMEM/F12) supplemented with 100 µg/mL penicillin-streptomycin, 125 U/mL DNase I, and 1X collagenase/hyaluronidase at 37 °C for 30 min. The minced tumor tissue solution was centrifuged at 1500 rpm for 3 min and washed thrice with RPMI medium to remove the enzyme solution. The cell pellet was resuspended in the culture medium and filtered using a 40 μm strainer. The proposed mechanical dissociation uses physical force to separate the tumor tissue into cells. After placing the minced tissue on the strainer, it was gently pressed and ground onto the membrane using a 1 mL syringe plunger. The cells remaining in the strainer were collected by washing the membrane with an RPMI medium. The cells were centrifuged at 1500 rpm for 3 min. The cell suspension was passed through a 40 μm strainer and centrifuged at 1500 rpm for 3 min. The pellet containing PDCs was collected and cultured in advanced DMEM/F12 medium along with supplements for PDO culture. Further information on the supplements has been provided in Table S3. The isolated PDCs were separated into cancer cells by Percoll-gradient centrifugation, as described above. A part of the obtained PDC was cryopreserved at 5 × 10^5^ cells per 1 mL of Cellbanker 2 solution, and the remaining samples were subjected to PDO culture and drug screening analysis. Table S4 includes the PDO culture and drug screening assay success rates for total enrolled lung cancer patients. In addition, information on patient-derived biological samples and the viability and total cell number of PDCs are listed.

### Experimental protocol of histology and immunostaining

Additional quality control (QC) samples were prepared to verify whether the PDOs retained oncological characteristics similar to those of the primary tumor. The prepared PDOs for QC were subjected to immunohistochemistry (IHC) analysis for pathological reading similar to the patient tissue (Fig. 2K). The isolated PDCs were seeded in a 4-well plate at a concentration of approximately 1 × 10^5^ cells and 70% Matrigel (70 v/v) per 30 µL volume. The plate was inverted and placed at 4 °C for 5 min. The 4-well plate containing the PDCs was transferred and gelled for 30 min at 37 °C in a humidified incubator containing 5% CO_2_. The cells were cultured for approximately two weeks until the PDOs were formed; the culture medium was changed every three days. PDOs were fixed, embedded in paraffin, sectioned and stained. TTF-1, napsin A, p63 and CK7 were stained using Bond-III stainer (Leica Microsystems). p40, ALK(D5F3) and PD-L1(SP263) staining was performed using BenchMark ULTRA IHC/ISH System (Roche Diagnostics). PD-L1(22C3) staining was performed using Autostainer Link 48 (Agilent). Hematoxylin and eosin (H&E) and immunohistochemical images were acquired using an Ocus®40 digital microscope scanner (Grundium Oy, Finland). Samples were confirmed as tumor tissue on the basis of histopathological assessment. The diagnosis of each case was confirmed by pathologists at Seoul St. Mary’s Hospital (The Catholic University).

### Fluorescence in situ hybridization (FISH) analysis for detecting ALK rearrangement

FISH analysis was examined using the Vysis ALK Break Apart FISH Probe Kit (Abbott Molecular, Chicago, IL, USA). Paraffin sections from patient-tumor and organoids blocks in 5µm thickness were prepared for FISH staining and FISH analysis was carried out as a description in a test kit insert. Paraffin sections were deparaffinized using xylene and were dehydrated with graded ethanol. Dehydrated sections were incubated at 0.2 N HCl for 20min and were washed using protease solution for 30min. Finally, these sections were fixed in 10% neutral buffered formalin (NBF). The slides treated with the ALK probe cocktail were incubated at 73°C for 7 min using Thermobrite (Abbott Molecular, Chicago, IL, USA) and then were incubated overnight at 37°C. The slides were washed twice using 2Xssc/0.3% NP-40 solution and were counterstained with DAPI. A positive case was defined as a case in which the 5’ green probe and the 3’ red probe exhibited split signals separated by more than the diameter of the two signals. Samples were evaluated as negative if < 5 out of 50 cells were identified as positive for ALK rearrangement and positive if > 25 out of 50 cells were identified as positive for ALK rearrangement by defined signal enumeration criteria. Samples that tested positive with 5–25 out of 50 cells were reconfirmed using additional 50 cells of the tumor and considered positive if the average of positive cells in the two assessments was ≥ 15% of tumor cells. The ALK-FISH evaluation of each case was confirmed by pathologists at Seoul St. Mary’s Hospital (The Catholic University).

### Next generation sequencing (NGS) analysis

Genomic DNA (gDNA) was extracted from formalin-fixed, paraffin-embedded (FFPE) lung cancer tissues or PDOs using a Maxwell RSC DNA FFPE Kit (Promega, USA). gDNA concentration was quantified using Qubit™ fluorometer (Thermo Fisher Scientific, USA). The library preparation was performed using Oncomine Comprehensive Assay Plus System (Thermo Fisher Scientific, USA). The prepared library loaded onto Ion 550 chips (Thermo Fisher Scientific, USA) was processed using the Ion Chef system (Thermo Fisher Scientific, USA) and was sequenced on the Ion torrent S5 XL™ sequencer (Thermo Fisher Scientific, USA). Data were analyzed through Ion Reporter™ software (v5.20) (Thermo Fisher Scientific, USA).

## Results

### Optimization of PDC isolation method and pathological biomarkers similarity verification of PDO

**Fig. 3:**
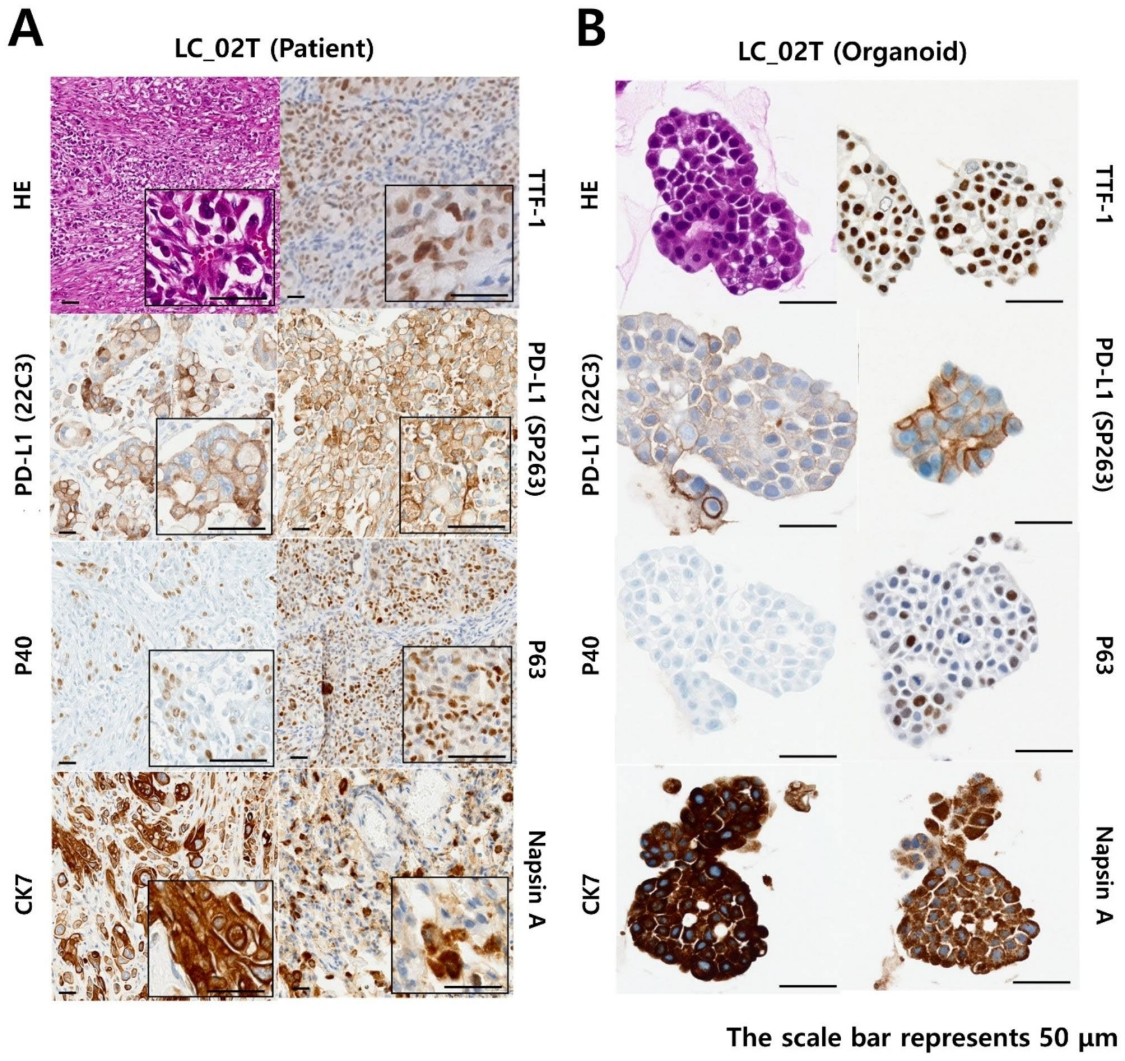
H&E and immunohistochemical staining images of patient-derived lung cancer tumor tissues and lung cancer PDOs. Results of IHC analysis using cancer tissues (**A**) and organoids (**B**) derived from the same patient with lung cancer; in lung cancer organoids, the expression patterns of lung cancer characteristic markers (TTF-1, P63, CK7 and Napsin A) were well maintained, the same as in patient lung cancer tissues

**Fig. 4:**
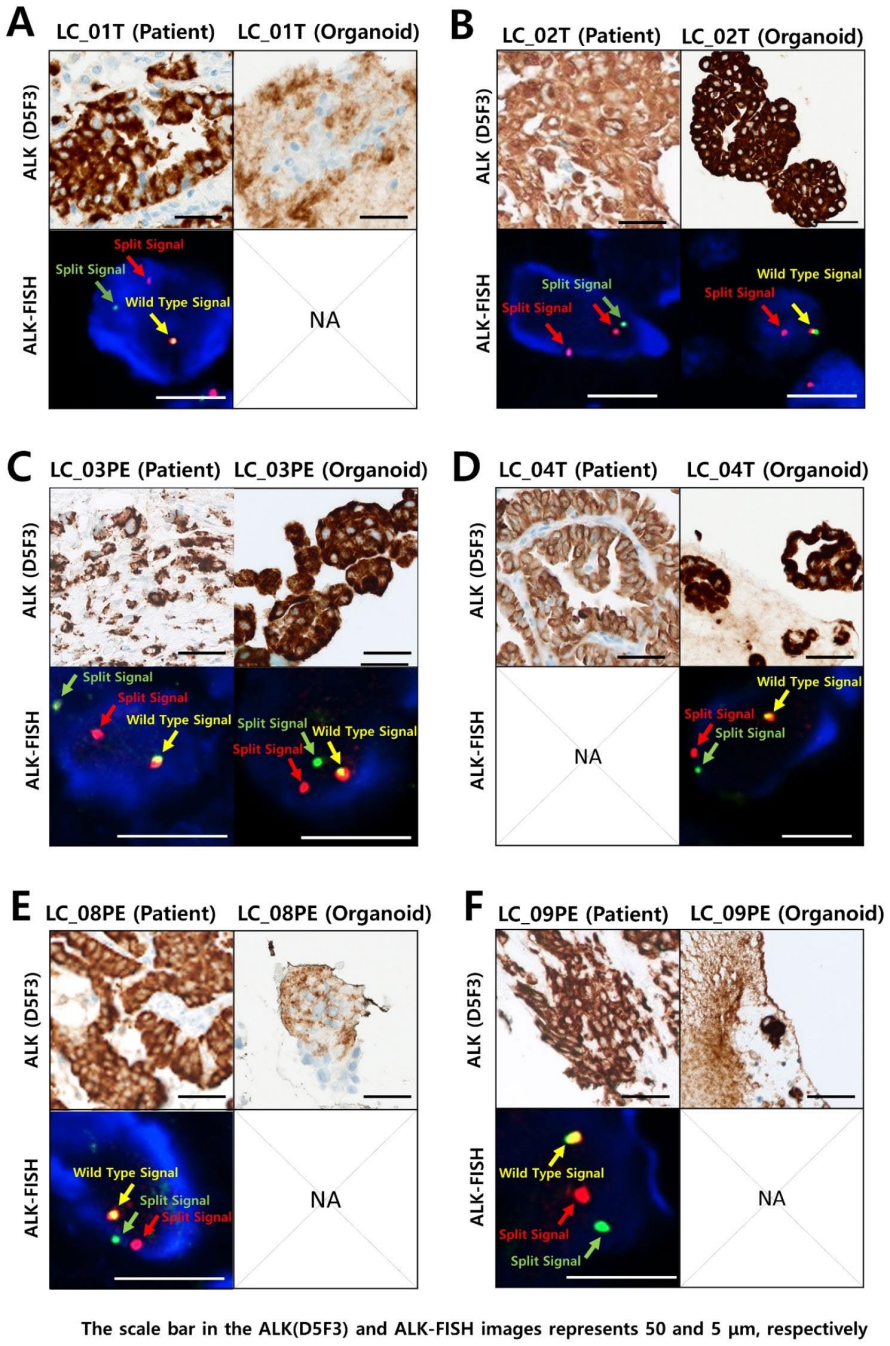
Immunohistochemical staining and ALK-Fluorescence In Situ Hybridization Evaluation (FISH) scanning images of patient-derived lung cancer tumor tissues and lung cancer PDOs. (**A - F**) IHC analysis of tumor tissue and PDO from ALK-positive patients (LC_01T, 02T, 03PE, 04T, 08PE and 09PE) verified similar ALK-positive expression patterns, respectively. Fluorescence In Situ Hybridization Evaluation (FISH) evaluation of ALK-EML4 break apart/split signal is the gold standard to investigate ALK rearrangement status. Scanned images showing fused red/green signals representing normal copies of the ALK (yellow arrows) and single red and green signals (red and green arrows) indicating that chromosomal break occurred between the 3 and the 5 contigs. ALK-EML4 break apart was verified in patient tissue (analysis was not performed in LC_04T patient), and ALK-EML4 break apart was also verified in PDO derived from ALK-positive patients (analysis was not performed in LC_01T, 08PE and 09PE)

To implement PDOs, PDCs were isolated from MPEs using Percoll-gradient centrifugation according to the procedures mentioned in Methods. Two tumor tissue dissociation methods were used to increase the yield of PDCs (Figure S2). Two PDCs were isolated from patient’s tumor tissues with lung cancer by enzymatic and mechanical dissociation methods and then cultured for three days. The difference in the growth rate of PDOs according to the dissociation method of patient-derived tumor tissue was quantitatively analyzed by image scanning (Figure S2A). The isolated PDCs were further unicellularized under enzymatic dissociation conditions. The PDCs isolated by the mechanical dissociation method formed mature PDOs after three days of incubation. Considering the variation in the initial number of PDCs seeded, the proliferation rate of PDO, according to dissociation conditions, was quantitatively analyzed based on the normalized area value of PDO after one day of incubation (Figure S2B). As a result, the PDO growth rate analyzed by approximately 1.3-fold in three days under enzymatic dissociation conditions but increased approximately 3.1-fold under mechanical dissociation conditions, suggesting that mechanical tumor tissue dissociation is a suitable method for the formation of mature PDO at a higher growth rate. The PDOs were prepared from patient-derived tumor tissue and MPE. The patient’s tumor tissues and PDOs were observed to maintain primary cancer characteristics through IHC analyses (Fig. 3A). The expression patterns of lung adenocarcinoma (LUAD) markers, such as thyroid transcription factor-1 (TTF-1), cytokeratin 7 (CK7), and napsin A, were maintained in PDO (Fig. 3B). The transformation-related protein 63 (p63), commonly known as a squamous cell carcinomas (SCC) marker, was expressed in the tumor tissues and PDO of patients with LC_02T. Immunohistochemically, it has been reported that p63 staining is mainly expressed in 87.5% of SCC, but also in 4.3% of LUAD [29]. ALK expression patterns were observed in the cytoplasm of tumor tissues and PDOs from all 6 patients positive for ALK rearrangement (Fig. 4A – 4F). The results of the IHC analysis of tumor tissues from patients with positive ALK rearrangements are listed in Table S5. In addition, ALK-FISH evaluation was performed on 5 cases of ALK-positive patient tissue and 3 cases of PDO that were confirmed to be ALK-positive through IHC analysis (Fig. 4A – 4F). In all tested tumor tissues and PDOs, the split signal of the 5’ green probe, 3’ red probe, and the yellow wild type signal were scanned. The ALK rearrangement-positive diagnosis was confirmed at similar levels in all patient tissues and PDOs. Unfortunately, ALK-FISH evaluation could not be performed in some cases due to difficulties in obtaining additional patient tissue (LC_04T) and PDO (LC_01T, 08PE and 09PE) samples. As a result of NGS analysis, patient LC_02T was EML-ALK variant 2, and LC_04T and LC_09PE were EML-ALK variant 3 (Table S5). EML4-ALK variant 1 (E13:A20) was the predominant variant type, followed by EML4-ALK variant 3 (E6:A20) and variant 2 (E20:A20). And it has been reported that EML4-ALK variant 3 were associated with worse prognosis [30].

### Comparison of AUC and CODRP index for drug sensitivity analysis

**Fig. 5:**
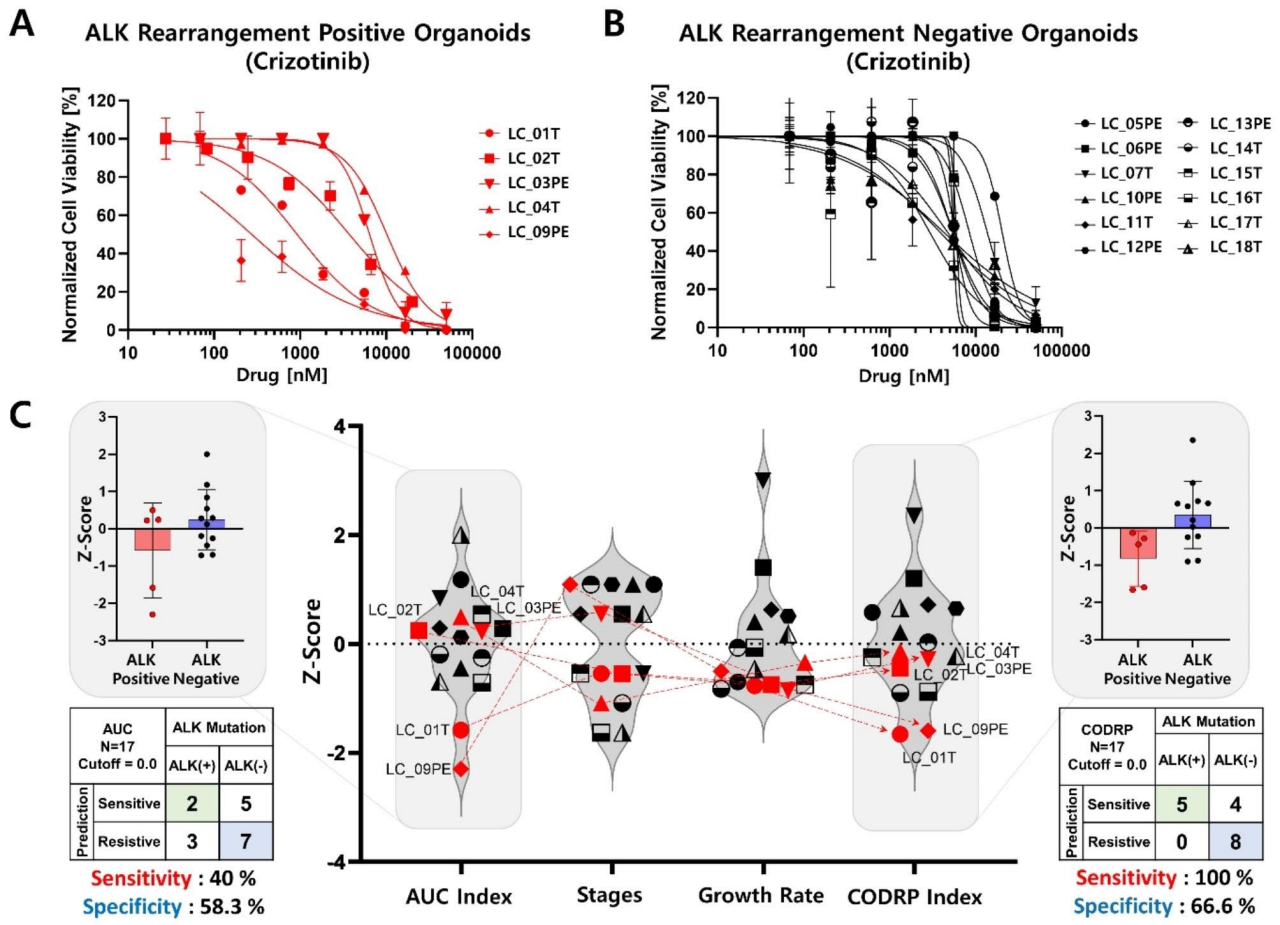
Lung cancer PDO-based high-throughput screening (HTS) and cancer organoid-based diagnosis reactivity prediction (CODRP) index analysis to crizotinib. DRC-based drug response analysis using ALK-positive (**A**) and ALK-negative (**B**) patient-derived organoids to crizotinib. (**C**) Comparative analysis of drug response based on the AUC and CODRP indices for crizotinib; The CODRP index is calculated as a Z-score value based on different mean and standard deviation (SD) values of crizotinib for individual PDO, considering the AUC index, cancer stage, and PDO growth rate through multiple linear regression (Mean, SD, and sample number of Crizotinib: 0.71, 0.26, 17). In the conventional AUC index-based analysis for the crizotinib, the sensitivity was 40% and the specificity was 58.3%, but as a result of the CODRP index-based drug sensitivity analysis, the analysis performance improved to 100% sensitivity and 66.6% specificity

**Fig. 6:**
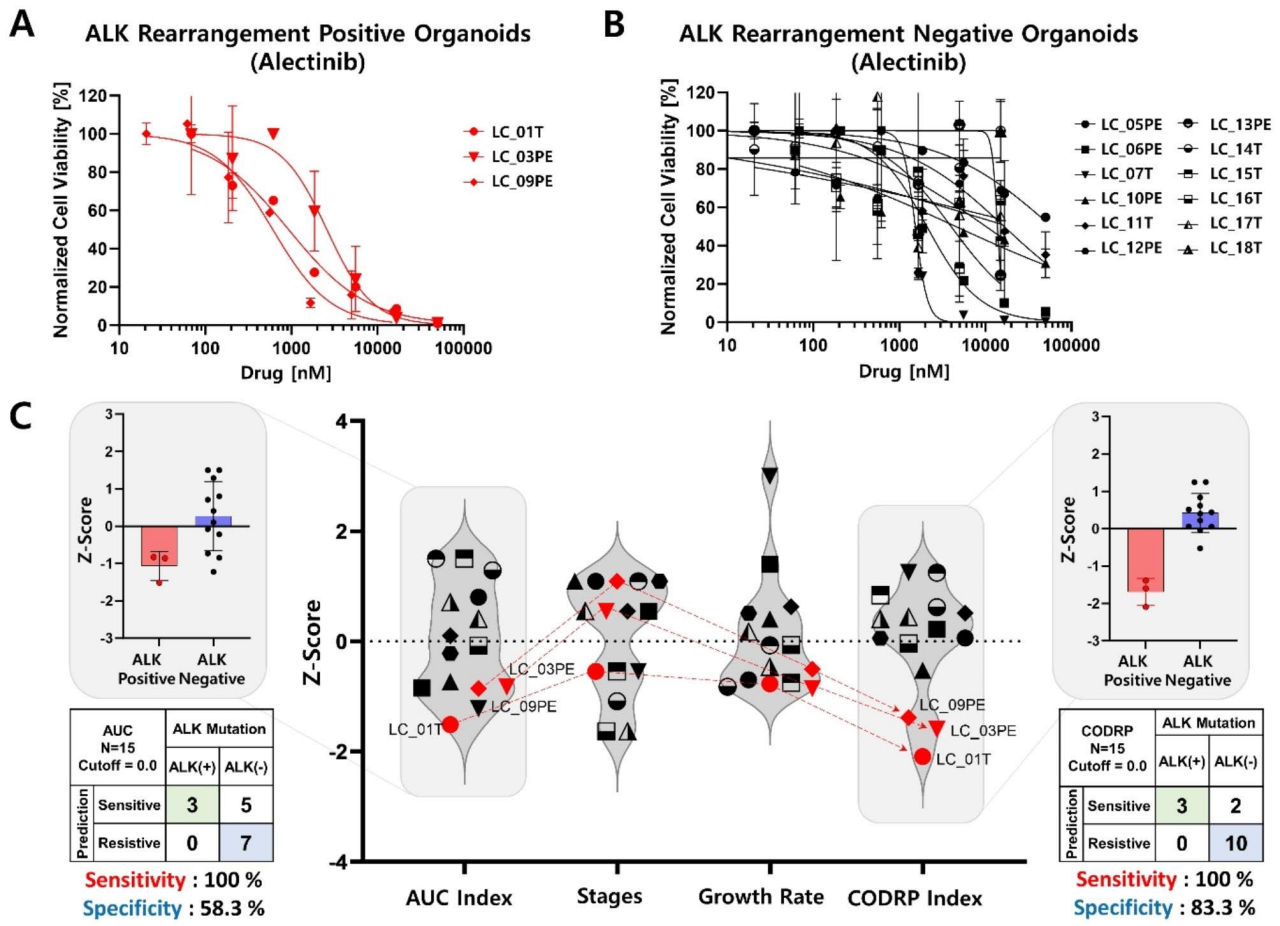
Lung cancer PDO-based high-throughput screening (HTS) and cancer organoid-based diagnosis reactivity prediction (CODRP) index analysis to alectinib. DRC-based drug response analysis using ALK-positive (**A**) and ALK-negative (**B**) patient-derived organoids to alectinib. (**C**) Comparative analysis of drug response based on the AUC and CODRP indices for alectinib; The CODRP index is calculated as a Z-score value based on different mean and standard deviation (SD) values of alectinib for individual PDO, considering the AUC index, cancer stage, and PDO growth rate through multiple linear regression (Mean, SD, and sample number of alectinib: 0.8, 0.36, 15). In the conventional AUC index-based analysis for the alectinib, the sensitivity was 100% and the specificity was 58.3%, but as a result of the CODRP index-based drug sensitivity analysis, the analysis performance improved to 100% sensitivity and 83.3% specificity

**Fig. 7:**
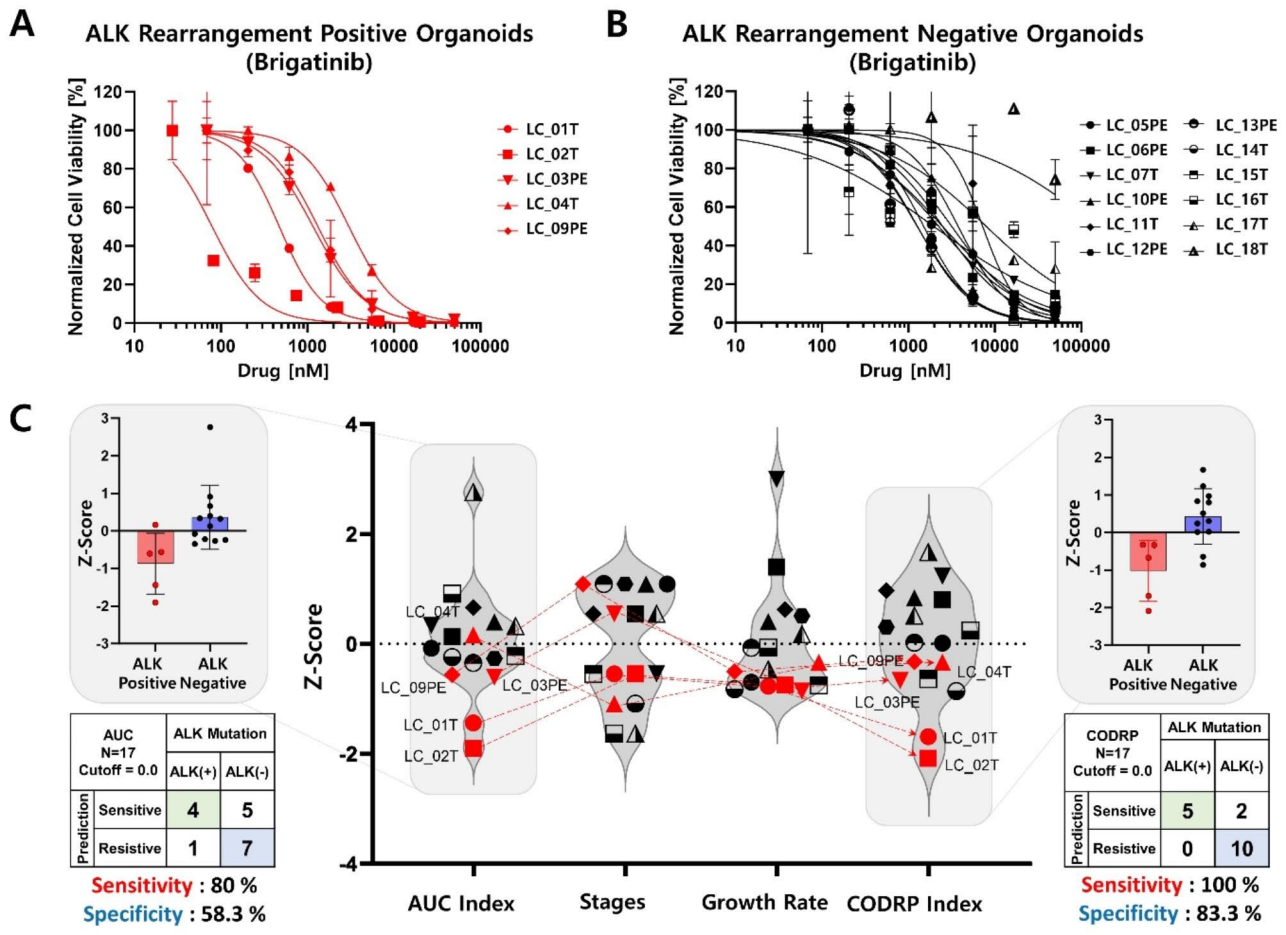
Lung cancer PDO-based high-throughput screening (HTS) and cancer organoid-based diagnosis reactivity prediction (CODRP) index analysis to brigatinib. DRC-based drug response analysis using ALK-positive (**A**) and ALK-negative (**B**) patient-derived organoids to brigatinib. (**C**) Comparative analysis of drug response based on the AUC and CODRP indices for brigatinib; The CODRP index is calculated as a Z-score value based on different mean and standard deviation (SD) values of brigatinib for individual PDO, considering the AUC index, cancer stage, and PDO growth rate through multiple linear regression (Mean, SD, and sample number of brigatinib: 0.71, 0.32, 17). In the conventional AUC index-based analysis for the brigatinib, the sensitivity was 80% and the specificity was 58.3%, but as a result of the CODRP index-based drug sensitivity analysis, the analysis performance improved to 100% sensitivity and 83.3% specificity. The cut-off value for classifying drug responses into sensitive and resistant groups to three ALK-targeted drugs is 0

Drug sensitivity test was performed using PDOs of positive and negative patients with ALK rearrangement to analyze the difference in drug sensitivity according to ALK rearrangement status towards the three ALK-targeted drugs (crizotinib, alectinib, and brigatinib) (Figs. 5, 6 and 7). Drug sensitivity for ALK-targeted drugs was quantitatively analyzed using the AUC values of the DRCs in ALK-positive (Figs. 5A and 6 A) and -negative (Figs. 5B, 6B and 7B) PDOs, respectively. Since the AUC value signifies an absolute drug efficacy value, the AUC index represents the relative efficacy in the test patient population including ALK-positive and -negative patients. The red dots in the graph represent ALK rearrangement-positive PDOs (Figs. 5C and 6C). When analyzing drug sensitivity based on the AUC index, the sensitivity and specificity of predicting ALK-targeted drugs according to ALK rearrangement status were evaluated. The sensitivity and specificity of crizotinib were analyzed as 40% and 58.3% (Fig. 5C), alectinib was 100% and 58.3% (Fig. 6C), and brigatinib was 80% and 58.3% (Fig. 7C). The alectinib drug could not be tested in LC_02T and LC_04T PDOs due to an unfortunate human error involving the use of incorrect alectinib drug stock. Because all 48 PDOs that passed QC were not banked into frozen stock and these PDOs (LC_02T and 04T) stock did not remain, additional drug screening analysis could not be performed. Despite being ALK rearrangement-positive PDOs, the drug efficacy analyses based on the AUC index were not well classified as sensitive responder groups compared to ALK rearrangement-negative PDOs for all three ALK-targeted drugs. As it is difficult to distinguish the response of targeted drugs specific to an oncogenic marker when analyzing drug efficacy by considering only the AUC index value, a drug response analysis method based on the CODRP index using multi-parameter analyses was proposed (Figs. 5C and 6C). CODRP algorithm used multi-parameters such as individual patient’s cancer stage and organoid growth as well as the AUC of the DRC. Therefore, the mean and standard deviation (SD) of the CODRP values were calculated for each drug. The drug response of each PDO was analyzed by the CODRP index, which is the Z-score of the CODRP values. Drug-sensitive and drug-resistant groups were classified based on the CODRP index value of 0. The sensitivity and specificity of predicting ALK-targeted drugs according to ALK rearrangement status were evaluated using the CODRP index. The sensitivity and specificity of crizotinib were analyzed as 100% and 66.6% (Fig. 5C), alectinib was 100% and 83.3% (Fig. 6C), and brigatinib was 100% and 83.3% (Fig. 7C). Improved sensitivity and specificity were verified compared to the conventional AUC index-based drug sensitivity analysis. When the response to ALK-targeted drugs was analyzed using the CODRP index, all five ALK rearrangement-positive PDOs were analyzed as sensitive responder groups to the three ALK-targeted drugs and they were well classified.

### Comparison of CODRP index-based drug sensitivity test and clinical treatment results

**Fig. 8:**
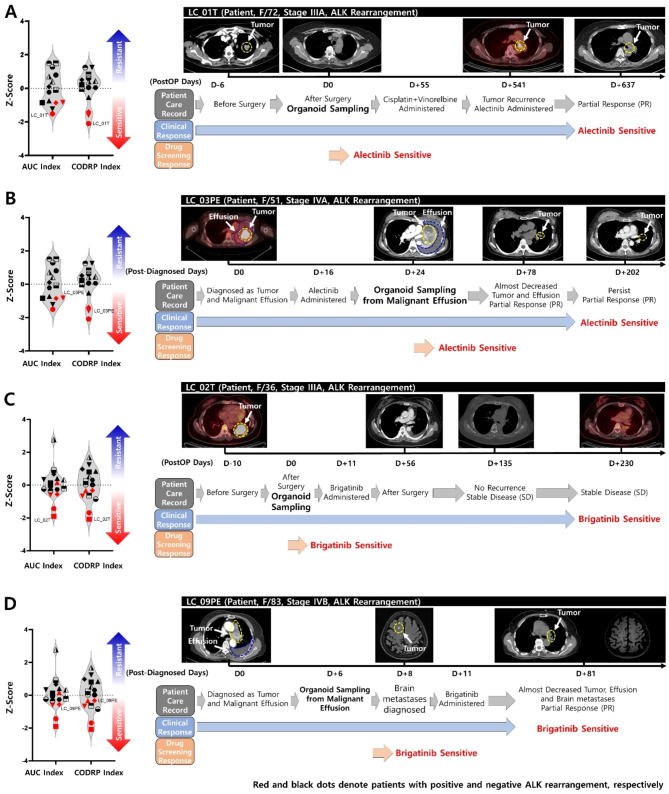
Clinical relevance of lung cancer PDO-based HTS analysis and CODRP index analysis. (**A**) The LC_01T patient was prescribed alectinib after the tumor recurred and showed a partial response (PR), and as a result of drug sensitivity test using LC_01T organoid derived from tumor tissue, showed a sensitive response to alectinib. (**B**) After being diagnosed with lung cancer accompanied by MPE, the LC_03PE patient was prescribed alectinib and showed PR; a drug sensitivity test using LC_03PE organoid derived from MPE showed a sensitive response to alectinib. (**C**) LC_02T patient was prescribed brigatinib after tumor tissue removal surgery and maintained stable disease (SD) without recurrence; drug sensitivity tests using LC_02T organoid derived from tumor tissues showed a sensitive response to brigatinib. (**D**) After being diagnosed with lung cancer accompanied by MPE and brain metastases, the LC_09PE patient was prescribed brigatinib and showed PR; a drug sensitivity test using LC_03PE organoid derived from MPE showed a sensitive response to brigatinib

We evaluated the response of patients to drug treatment by clinicians based on the Response Evaluation Criteria in Solid Tumors (RECIST 1.1) guidelines [31, 32]. RECIST criteria are based on the sum of the maximum diameters of the target lesions seen in imaging. RECIST 1.1 guidelines were evaluated as follows: complete/partial response (CR/PR), complete disappearance of all targets/greater than 30% decrease; stable disease (SD), change between − 30% and + 20%; and progressive disease (PD), greater than 20% increase. Previously, we performed drug sensitivity analysis with AUC and CODRP index using PDOs. The CODRP index is an analysis of drug sensitivity by considering the cancer diagnosis stage and PDO growth rate as additional factors to the AUC index, and these results of drug sensitivity analysis were compared with the drug treatment results of the same clinical patient as the origin of PDOs. Thus, AUC and CODRP index are calculated simultaneously in the same PDO. In LC_01T patient with lung cancer, the tumor recurred despite receiving platinum-doublet chemotherapy after tumor removal surgery and was prescribed the ALK-targeted drug alectinib, showing a partial response (PR) in which the tumor size decreased (Fig. 8A). Therefore, LC_01T patient was clinically classified as sensitive to alectinib. The drug sensitivity test results using the patient’s surgical tumor tissue-derived PDO (LC_01T) were classified as a sensitive group to alectinib, consistent with the clinical patient treatment results (Fig. 8A). In the LC_03PE patient, tumor tissue accompanied by MPE was prescribed alectinib, following which almost all tumors and MPE were reduced, showing PR (Fig. 8B). LC_03PE patient showed continuous partial response (PR) and was classified as a sensitive group to alectinib; the results of drug response analysis using PDO (LC_03PE) derived from MPE were classified as sensitive to alectinib, consistent with clinical patient treatment results (Fig. 8B). LC_02 patient was prescribed brigatinib following the tumor removal surgery and was classified as clinically sensitive to brigatinib as it showed stable disease (SD) with no tumor recurrence (Fig. 8C). In addition, it was classified as sensitive to brigatinib based on the drug response analysis conducted using surgically removed tumor tissue-derived PDO (LC_02T), which was consistent with clinical patient treatment results (Fig. 8C). In the LC_09PE patient, tumor tissue accompanied by MPE and brain metastases was prescribed brigatinib, following which almost all tumors and MPE were reduced, showing PR (Fig. 8D). LC_09PE patient showed continuous partial response (PR) and was classified as a sensitive group to brigatinib; the results of drug response analysis using PDO (LC_09PE) derived from MPE were classified as sensitive to brigatinib, consistent with clinical patient treatment results (Fig. 8D). Compared to the conventional AUC index, the CODRP index better classifies ALK-positive and negative PDO into sensitive and resistant groups, consistent with clinical patient treatment outcomes. The clinical information of all patients with positive and negative ALK rearrangements has been summarized in Table S6.

### Feasibility evaluation of CODRP Index-based Drug Sensitivity Test

**Fig. 9:**
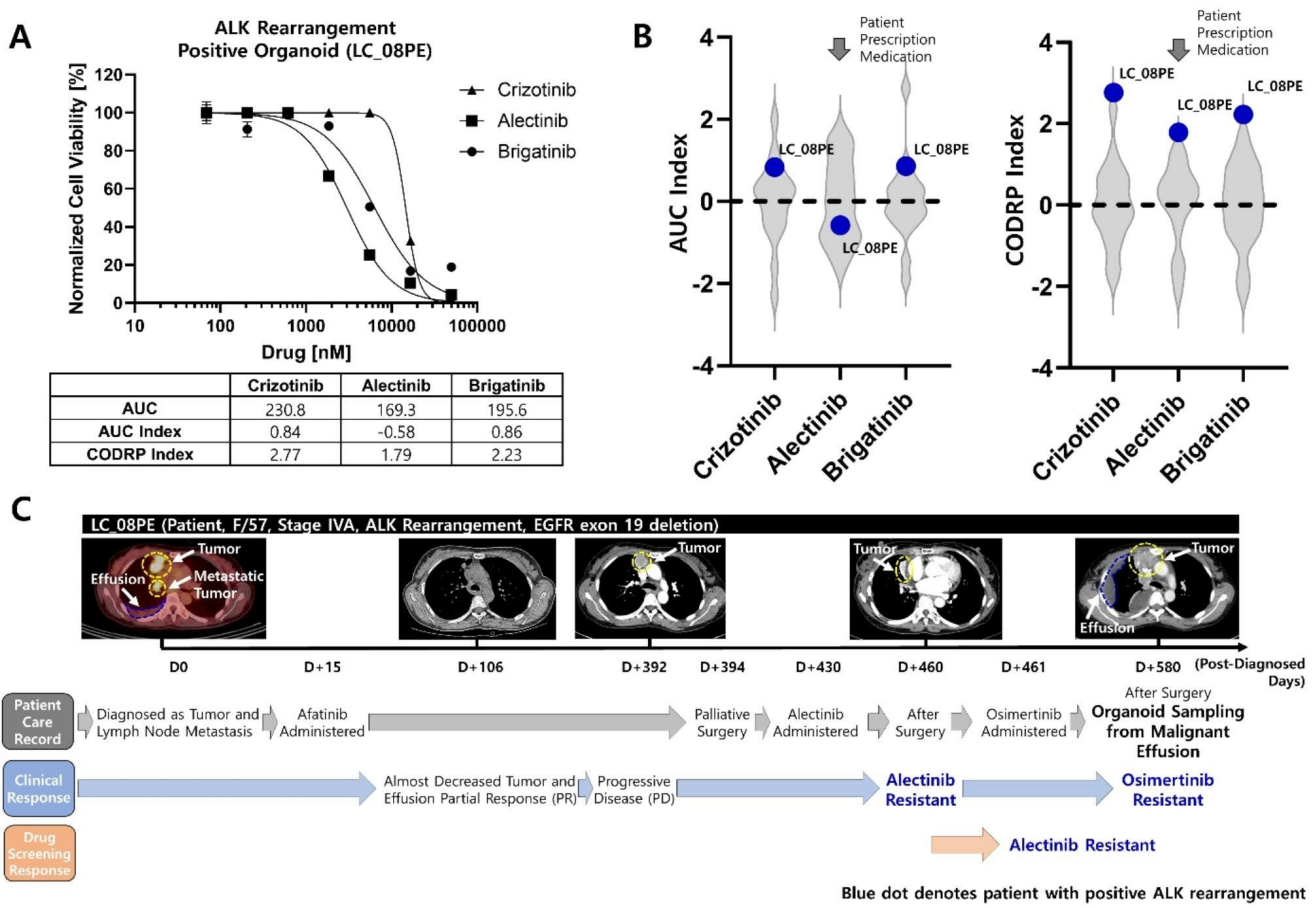
Evaluation of CODRP index-based drug sensitivity prediction platform by applying ALK-targeted drug-resistant patient sample. (**A**) Drug sensitivity test of three ALK-targeted drugs using LC_08PE organoid derived from patients with positive ALK. (**B**) The results of the drug sensitivity test based on the conventional AUC index predicted sensitive drug response to alectinib; in the proposed CODRP index-based drug sensitivity test, all three ALK-targeted drugs were predicted as a resistance group. (**C**) LC_08PE patient was prescribed alectinib after palliative surgery and showed resistance to alectinib; the drug sensitivity test using LC_08PE organoid derived from the MPE of LC_08PE patient showed resistance to alectinib

LC_08PE organoid was derived from the MPE of a patient with a positive ALK rearrangement, and a drug sensitivity test was performed for the three ALK-targeted drugs (Fig. 9A). The conventional drug sensitivity test based on the AUC index classified the ALK-targeted drug, alectinib, as a sensitive group with an AUC Z-score value of -0.58, whereas crizotinib and brigatinib were analyzed to have resistant drug responses with AUC Z-scores ranging from 0.84 to 0.86. However, in the CODRP index-based drug sensitivity test, drug sensitivity was predicted as a high resistance group with a CODRP index Z-score value of ≥ 1.79 for all three ALK-targeted drugs due to high cancer stage and rapid cell growth rate (Fig. 9B). LC_08PE patient was diagnosed as a tumor with MPE and lymph node metastasis and was resistant to the ALK-targeted drug alectinib, despite being positive for EGFR exon 19 deletion and ALK rearrangement. In particular, LC_08PE patient had showed partial response (PR) to afatinib, an EGFR-targeted drug, after the lung cancer diagnosis, leading to the disappearance of almost all the tumors. However, the tumor recurred and showed drug resistance despite surgery and treatment with alectinib and osimertinib (Fig. 9C).

## Discussion

In this study, we developed a disposable nozzle-type cell spotter and performed drug screening analysis for three ALK-targeted drugs using PDOs from patients with lung cancer. We have proposed the optimal mechanical dissociation method for isolating PDCs from tumor tissues and MPEs in patients with lung cancer. Subsequently, PDCs have been cultured in 3D using the proposed techniques to implement PDOs. PDOs must replicate the unique tumor characteristics of primary cancer [33, 34]. As a result of IHC analyses of tissues and PDO of a patient diagnosed with LUAD, enlarged cell nuclei and cytoplasmic structures were seen in the H&E [35, 36]. In addition, the expression patterns of LUAD markers [37, 38], such as thyroid transcription factor-1 (TTF-1), cytokeratin 7 (CK7), and napsin A, ALK expression patterns were maintained in PDO (Fig. 3A and B). The same ALK expression patterns were observed in the cytoplasm of tumor tissues from patients positive with ALK rearrangement. In addition, we have further validated whether the characteristics of ALK-positive patients are maintained in PDOs (Fig. 4A F). Fluorescence in situ hybridization (FISH) evaluation of the ALK-EML4 break apart/split signal is the gold standard for investigating ALK rearrangement status [39]. The ALK rearrangement-positive diagnosis was confirmed at similar levels in all patient tissues and PDOs (Fig. 4A F). Although FISH is considered the universally accepted reference standard, IHC has shown great sensitivity and specificity compared to FISH. D5F3 companion diagnostic assay is widely used as a standalone test to select NSCLC patients for ALK TKIs in practice [40], resulting in not all patient samples undergoing ALK-FISH analysis. Therefore, an optimal protocol for implementing PDOs was established by isolating PDCs from patient-derived tumor tissue and MPE, which successfully verified that the patient’s unique primary lung cancer characteristics were well maintained and reflected in the PDOs implemented by the proposed method.

**Fig. 10:**
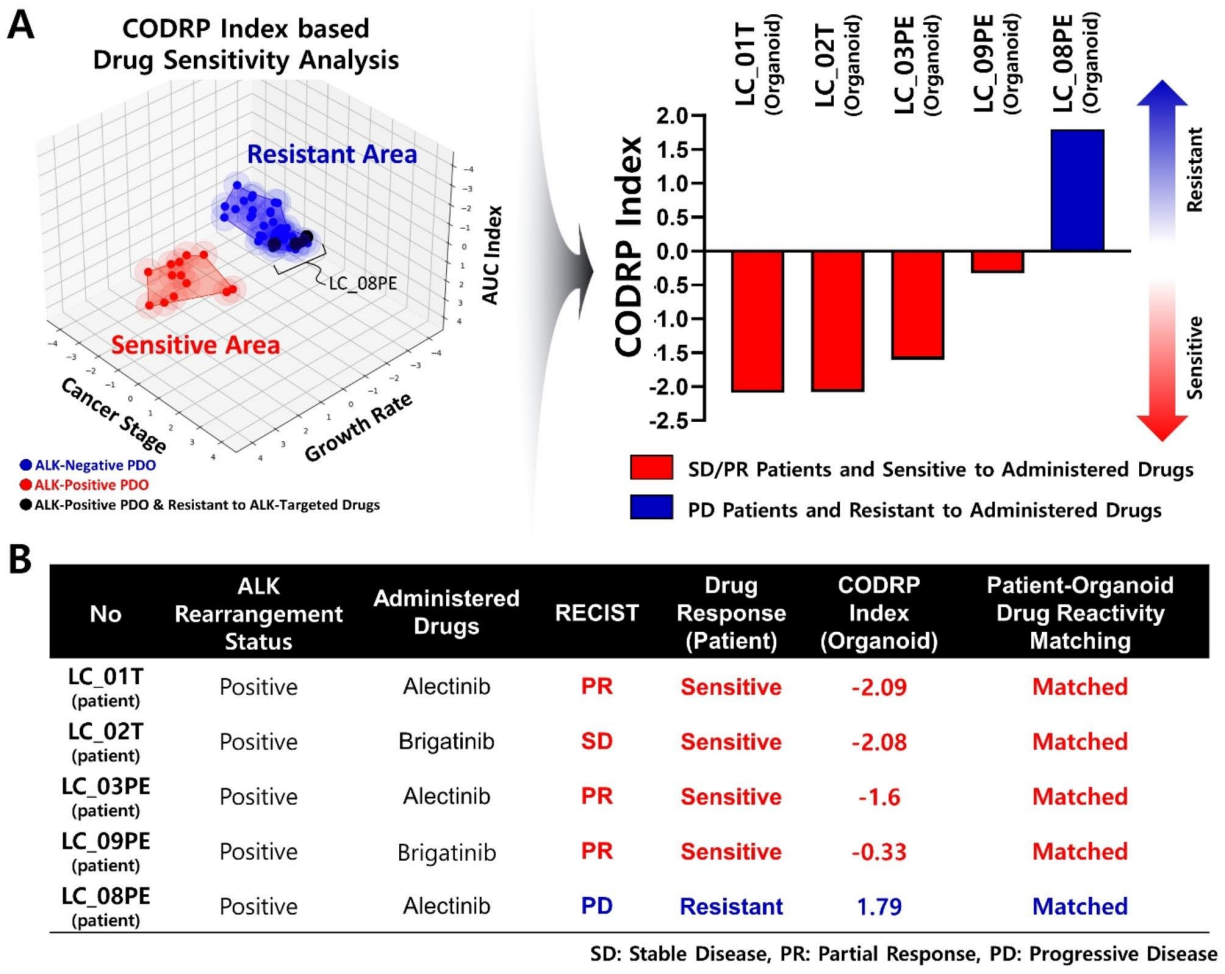
Summary of RECIST and CODRP index-based drug sensitivity test results. (**A**) Comparison of CODRP index-based drug sensitivity test and patient’s RECIST-based drug treatment response assessment results. (**B**) LC_01T, 02T, 03PE and 09PE patients showed effects on the prescribed drugs (alectinib and brigatinib), and sensitive drug responses were also analyzed in organoid models; LC_08PE patient had no therapeutic effect on the prescribed drug (alectinib) and showed drug resistance in an organoid model

Using these PDOs, we performed a drug sensitivity test to investigate the response of ALK-targeted drugs (Figs. 5, 6 and 7). We predicted that ALK-positive PDOs would be more sensitive to ALK-targeted drugs than ALK-negative PDOs. However, we found that PDO’s drug response affected cancer stage and cell growth. For example, fast-growing cells with a high cancer index show resistance to drugs. The drug sensitivity analysis, as determined by the AUC index, did not distinguish the sensitivity of ALK-targeted drugs based on ALK rearrangement status. Compared to ALK-negative PDOs, The LC_02T, 03PE, 04T showed higher drug resistance to crizotinib and LC_03PE, 09PE to alectinib and LC_04T to brigatinib. We observed that the growth rate was different for each PDO, and there was a negative correlation between the growth rate of the PDO and the AUC value representing drug efficacy. In addition, because the cancer progression status differs for each patient with lung cancer, the diagnosed lung cancer stages were scored [41] and reflected in drug sensitivity test in the CODRP index. Therefore, we developed a novel approach to drug sensitivity test of ALK-targeted drugs using a CODRP index, which incorporates both PDO growth rate and cancer diagnosis stage in addition to AUC values. Using the CODRP index, we analyzed the drug sensitivity of ALK-targeted drugs and successfully distinguished drug sensitivity based on ALK rearrangement status. As a result of the CODRP index-based drug sensitivity analysis, the sensitivity and specificity for all three ALK-targeted drugs were improved compared to the conventional AUC index-based analysis. Therefore, rather than analyzing the drug sensitivity of PDO by considering the AUC index as a single parameter of drug efficacy, the proposed drug sensitivity test platform based on the CODRP index improved the ALK-targeted drug efficacy analysis. Additionally, we used early passaged PDO derived from lung cancer patients for drug sensitivity analysis. Therefore, PDOs consider tumor cell population and tumor heterogeneity similar to tumor mosaicism in clinical lung cancer patients. To verify the clinical relevance of the previously performed drug response analysis results based on the CODRP index, we compared the correlation between the PDO-based drug sensitivity test and the results of drug response in clinical patients with lung cancer (Fig. 8). In summary, the drug sensitivity test applied with multi-parameter CODRP index analyses using PDOs was verified to be consistent with the clinical patient drug treatment response. Although it may take several months or more to determine a patient’s response to the drug, the proposed CODRP drug response analysis method can potentially predict a patient’s response to a variety of drugs approximately two weeks before starting drug treatment. The proposed CODRP index-based drug sensitivity test was applied to the seventeen previously accumulated PDO drug sensitivity data libraries and was performed for three ALK-targeted drugs (Fig. 9). Unlike another case, LC_08PE was already treated ALK-targeted drug and showed resistance. Therefore, we registered this case as a special case to validate the CODRP algorithm, even though sample acquisition was after ALK-targeted drug treatment (Fig. 9). Therefore, CODRP index-based drug sensitivity analysis also predicted rare cases of ALK-targeted drug resistance in ALK-positive patients. The effectiveness of the CODRP index-based drug sensitivity test, which differs from the conventional AUC-based drug sensitivity test results, was evaluated by comparing the clinical drug treatment response with the drug sensitivity test results performed using PDOs. In the CODRP index-based drug sensitivity analysis, the sensitive and resistant regions for ALK-targeted drugs were well distinguished (Fig. 10A and Figure S3). In particular, in the case of LC_08PE, which showed resistance to the ALK-targeted drug despite being positive for ALK rearrangement, as confirmed above, it was distributed in the region of the resistance group to the ALK-targeted drugs (Fig. 10A). Therefore, direct comparison with clinical patient drug treatment results verified that the CODRP index-based drug sensitivity test results showed a higher predictive feasibility for clinical drug treatment response. In this study, evaluation results of the clinical drug response for ALK-targeted drugs administered to 5 patients with positive ALK rearrangement were compared with the CODRP index-based drug sensitivity test results using PDOs (Fig. 10A). 4 patients with lung cancer showed a sensitive response to ALK-targeted drugs and were analyzed as a sensitive group in the CODRP index-based drug sensitivity test result, which matched well with the clinical drug treatment response. In addition, even in cases showing resistance to ALK-targeted drugs, despite being positive for ALK rearrangement, CODRP index-based drug sensitivity test results were analyzed as the same resistance group for ALK-targeted drugs. The corresponding results have been summarized in Fig. 10B.

## Conclusion

We implemented a PDO that can recapitulate patients with lung cancer in a 384-pillar/well plate and developed a disposable nozzle-type cell spotter to enable efficient HTS-based drug sensitivity tests. The proposed CODRP index value-based drug sensitivity test is a multiparameter analysis that considers the AUC index, PDO growth rate, and cancer diagnosis stage. The feasibility of the proposed model was verified for rare ALK mutation cases and ALK-targeted drugs by accumulating a lung cancer PDO library. In addition, the CODRP index value-based drug sensitivity test results were compared with the clinical drug treatment results and cross-validated. Therefore, the PDO-based HTS platform and the proposed CODRP index-based drug sensitivity test are suitable models for precision medicine and new drug discovery for patients with lung cancer.

### Electronic supplementary material

Below is the link to the electronic supplementary material.

**Supplementary Material 1: Figure S1**. Summary of Illustration and Limitations for the Conventional 3D-Cell Spotter. (**A**) A conventional stainless nozzle-type cell spotter that dispenses samples with the pressure of a syringe pump. A dead volume of 50 μL or more is required to dispense high-viscosity samples. In addition, a nozzle-washing process is required to prevent cross-contamination whenever a sample is changed. **Figure S2**. Comparison of Patient-derived Cell Preparation Methods by Enzymatic and Mechanical Dissociation. (**A**) Image of organoid culture and proliferation rate difference analysis according to enzymatic and mechanical dissociation methods. (**B**) Quantitative analysis of differences in lung cancer organoid growth rates according to different dissociation methods through 3D live cell-staining. **Figure S3**. Results of CODRP index-based drug sensitivity test for each ALK-targeted drug. Drug response to three types of ALK-targeted drugs; (**A**) crizotinib, (**B**) alectinib and (**C**) brigatinib was analyzed into sensitive and resistant areas according to the ALK status of PDOs. **Table S1**. Cancer stage scoring standard table and individual patient’s cancer stage, growth rate, AUC, AUC Index, and CODRP Index. **Table S2**. Scoring table of cancer stage for CODRP index. **Table S3**. Recipe of the lung cancer PDO culture media. **Table S4**. Summary information of lung cancer patient-derived samples and dissociated PDC (viability and total cell number). **Table S5**. Summary of pathology analysis. **Table S6**. Summary of clinical information. **Table S7**. Summary of multiple linear regression (MLR) analysis results

## Data Availability

All data generated or analysed during this study are included in this published article and its supplementary information files.

## Abbreviations

CODRP: Cancer organoid-based diagnosis reactivity prediction
NSCLC: Non-small cell lung cancer
PDOs: Patient-derived organoids
HTS: High-throughput screening
AUC: Area under the curve
ALK: Anaplastic lymphoma kinase
EML4: Echinoderm microtubule-associated protein-like 4
PDXs: Patient-derived xenografts
PS-MA: Polystyrene-co-maleic anhydride
MPE: Malignant pleural effusion
ECM: Extracellular matrix
DRC: Dose-response curve
IHC: Immunohistochemistry
PBS: Phosphate-buffered saline
DMSO: Dimethyl sulfoxide
RPMI: Roswell park memorial institute
ATP: Adenosine triphosphate
QC: Quality control
LUAD: Lung adenocarcinoma
TTF-1: Thyroid transcription factor-1
CK7: Cytokeratin 7
FISH: Fluorescence in situ hybridization
PR: Partial response
SD: Stable disease
PD: Progressive disease
HBSS: Hank’s balanced salt solution
FBS: Fetal bovine serum
EDTA: Ethylenediamine tetraacetic acid
H&E: Hematoxylin and eosin
DMEM/F12: Dulbecco’s modified eagle medium/nutrient mixture F-12
MLR: Multiple linear regression
