# Correction: Cancer organoid-based diagnosis reactivity prediction (CODRP) index-based anticancer drug sensitivity test in ALK-rearrangement positive non-small cell lung cancer (NSCLC)

**DOI:** 10.1186/s13046-023-02919-3

**Published:** 2023-12-06

**Authors:** Sang-Yun Lee, Hyeong Jun Cho, Jimin Choi, Bosung Ku, Seok Whan Moon, Mi Hyoung Moon, Kyung Soo Kim, Kwanyong Hyun, Tae-Jung Kim, Yeoun Eun Sung, Yongki Hwang, Eunyoung Lee, Dong Hyuck Ahn, Joon Young Choi, Jeong Uk Lim, Chan Kwon Park, Sung Won Kim, Seung Joon Kim, In-Seong Koo, Woo Seok Jung, Sang-Hyun Lee, Chang Dong Yeo, Dong Woo Lee

**Affiliations:** 1 Department of Biomedical Engineering, Gachon University, Seongnam, 13120 Republic of Korea; 2 Central R & D Center, Medical & Bio Decision (MBD) Co., Ltd, Suwon, 16229 Republic of Korea; 3 Division of Pulmonology, Department of Internal Medicine, Seoul St. Mary’s Hospital, College of Medicine, The Catholic University of Korea, Seoul, Republic of Korea; 4 Department of Thoracic and Cardiovascular Surgery, College of Medicine, The Catholic University of Korea, Seoul, 06591 Korea; 5 Department of Hospital Pathology, College of Medicine, The Catholic University of Korea, Seoul, 06591 Korea; 6 Division of Pulmonary and Critical Care Medicine, Department of Internal Medicine, Incheon St. Mary’s Hospital, College of Medicine, The Catholic University of Korea, Seoul, Republic of Korea; 7 Division of Pulmonary, Critical Care and Allergy, Department of Internal Medicine, Yeouido St. Mary’s Hospital, College of Medicine, The Catholic University of Korea, Seoul, Republic of Korea; 8 Department of Otorhinolaryngology-Head and Neck Surgery, College of Medicine, The Catholic University of Korea, Seoul, Republic of Korea; 9 Department of Biomedicine & Health Sciences, College of Medicine, The Catholic University of Korea, Seoul, Republic of Korea; 10 Postech-Catholic Biomedical Engineering Institute, College of Medicine, The Catholic University of Korea, Songeui Multiplex Hall, Seoul, Republic of Korea; 11 Division of Pulmonary and Critical Care Medicine, Department of Internal Medicine, Eunpyeong St. Mary’s Hospital, College of Medicine, The Catholic University of Korea, Seoul, Republic of Korea

**Correction:** ***J Exp Clin Cancer Res*** **42, 309 (2023)**

10.1186/s13046-023-02899-4

Following publication of the original article [1], authors would like to correct the Funding section of their article.

The sentence currently reads:

This research was supported by the Bio & Medical Technology Development Program of the National Research Foundation (NRF) funded by the Korean government (MSIT) (No. 2019M3A9H2032425). This work was supported by the National Research Foundation of Korea (NRF) grant funded by the Korean government (MSIT) (No. 2020R1I1A306655012). This work was supported by the Ministry of Science and ICT, (Project Number: (2022) ERIC07_1_1) and Commercialization Promotion Agency for R&D Outcomes (COMPA).

The sentence should read:

This research was supported by the Bio & Medical Technology Development Program of the National Research Foundation (NRF) funded by the Korean government (MSIT) (No. 2019M3A9H2032425). This research was supported by the Commercialization Promotion Agency for R&D Outcomes(COMPA) funded by the Ministry of Science and ICT(MSIT) (project number: RS_2023-00259649, No. 1,711,196,063).This work was supported by the Korea Medical Device Development Fund grant funded by the Korea government (the Ministry of Science and ICT; the Ministry of Trade, Industry and Energy; the Ministry of Health & Welfare; and the Ministry of Food and Drug Safety) (project number: RS_2023-00255627).

The correction does not affect the overall result or conclusion of the article. The original article has been corrected.
