# Supplementary material for: Cancer organoid-based diagnosis reactivity prediction (CODRP) index-based anticancer drug sensitivity test in ALK-rearrangement positive non-small cell lung cancer (NSCLC)
Source: J Exp Clin Cancer Res. 2023 Nov 22;42:309. doi: 10.1186/s13046-023-02899-4 (PMC10664561; doi:10.1186/s13046-023-02899-4)

**
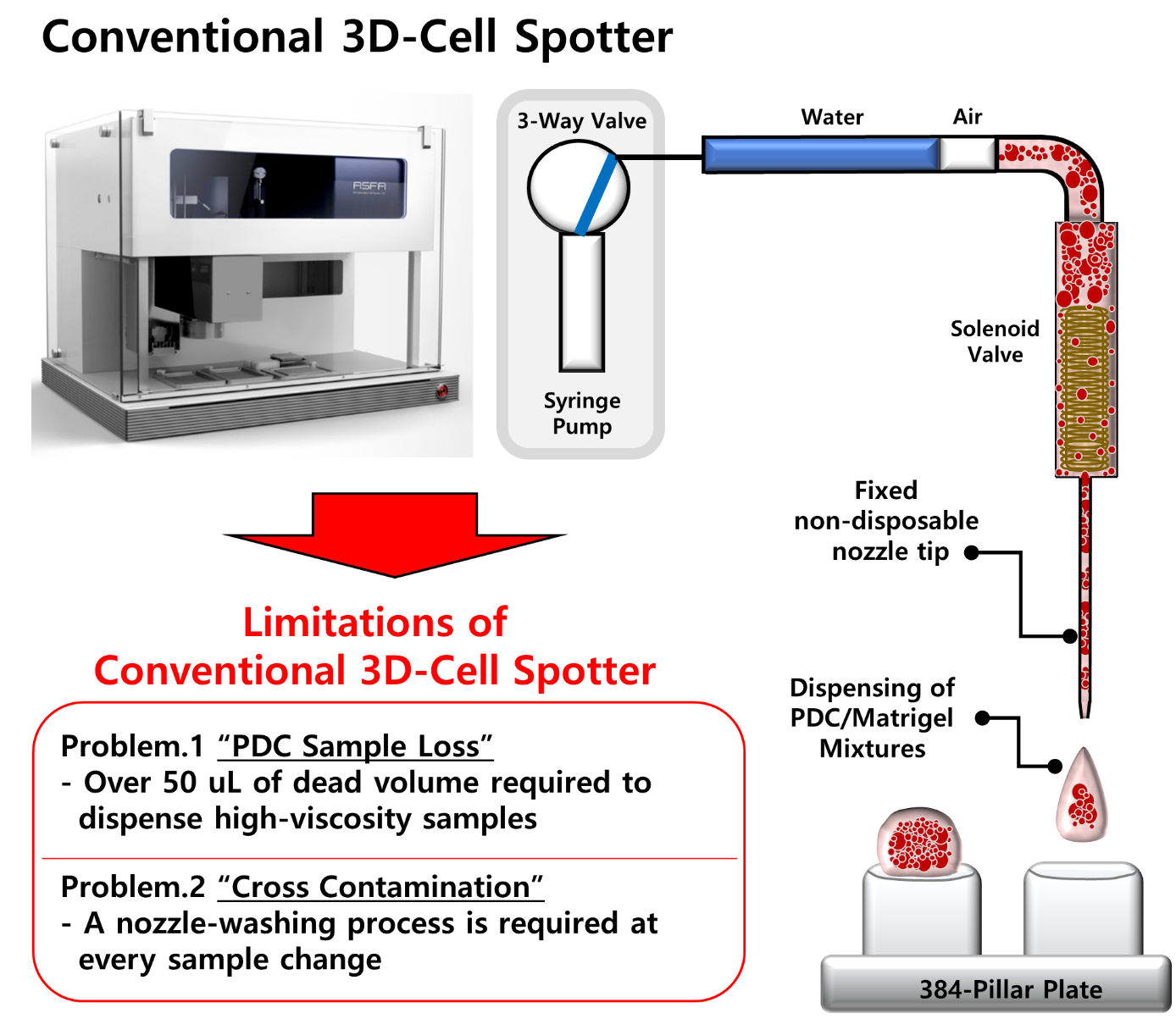
**

**Figure S1.** Summary of Illustration and Limitations for the Conventional 3D-Cell Spotter. (A) A conventional stainless nozzle-type cell spotter that dispenses samples with the pressure of a syringe pump. A dead volume of 50 μL or more is required to dispense high-viscosity samples. In addition, a nozzle-washing process is required to prevent cross-contamination whenever a sample is changed.

**
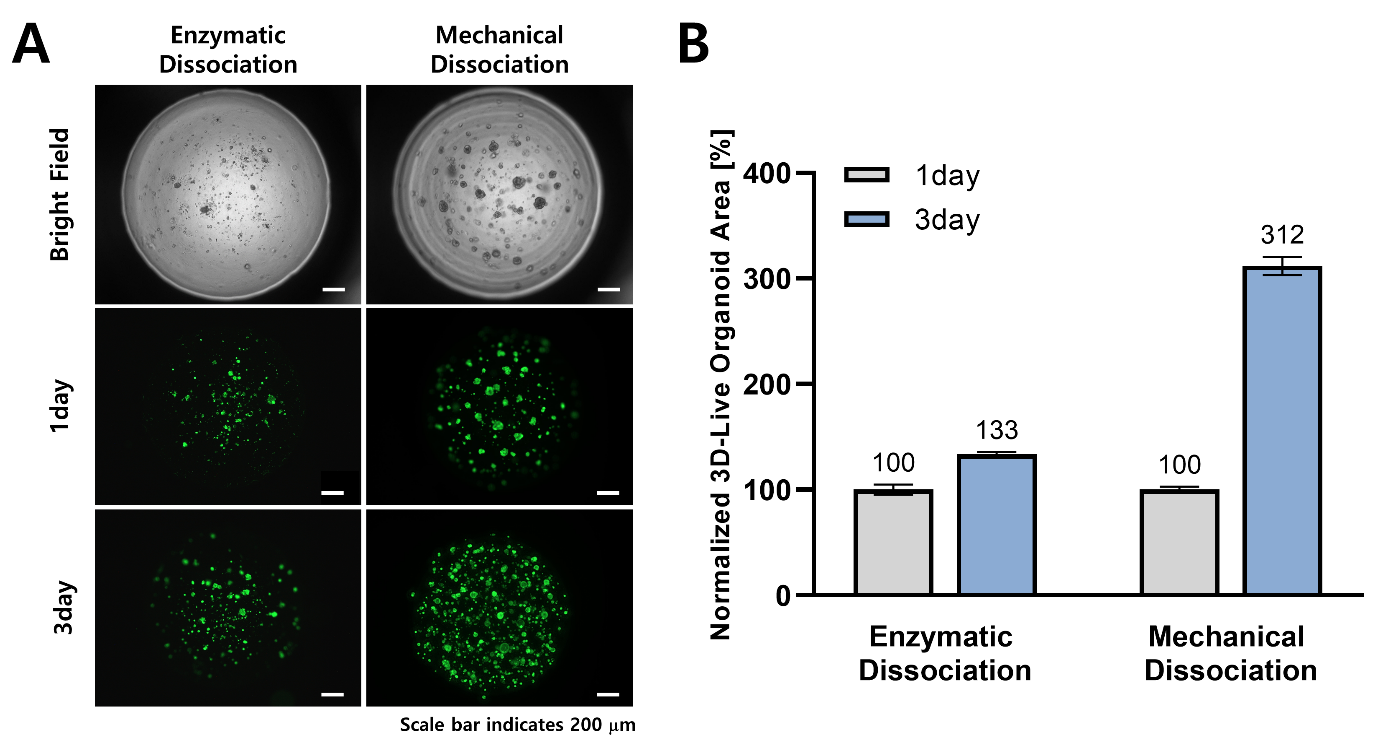
**

**Figure S2.** Comparison of Patient-derived Cell Preparation Methods by Enzymatic and Mechanical Dissociation. (A) Image of organoid culture and proliferation rate difference analysis according to enzymatic and mechanical dissociation methods. (B) Quantitative analysis of differences in lung cancer organoid growth rates according to different dissociation methods through 3D live cell-staining.

**
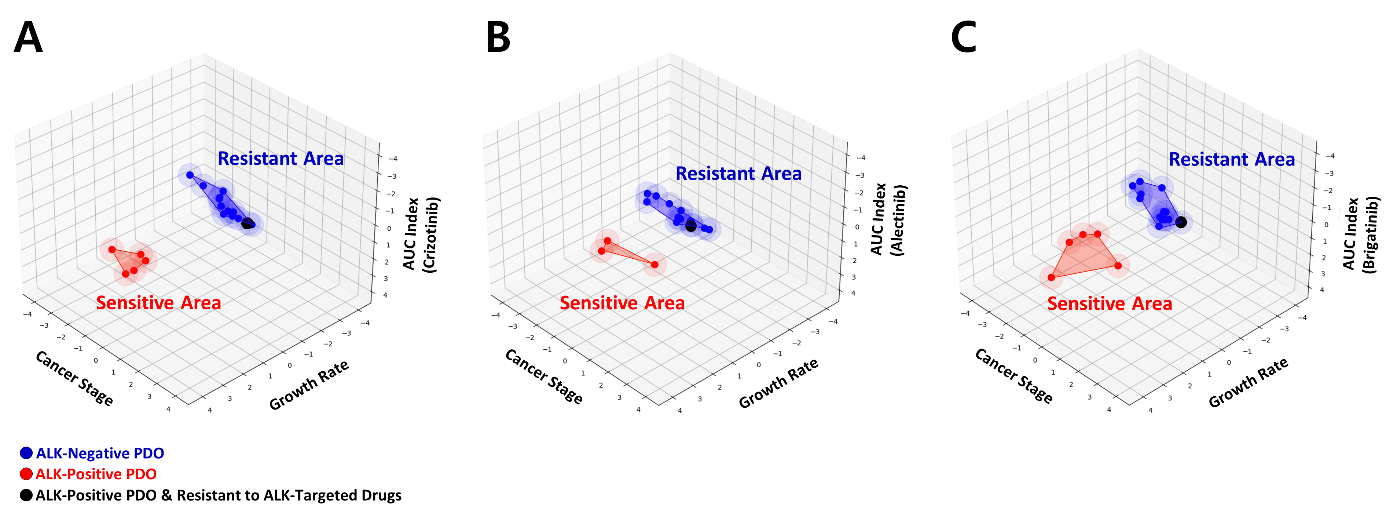
**

**Figure S3.** Results of CODRP index-based drug sensitivity test for each ALK-targeted drug. Drug response to three types of ALK-targeted drugs; (A) crizotinib, (B) alectinib and (C) brigatinib was analyzed into sensitive and resistant areas according to the ALK status of PDOs.

**Table S1.** Cancer stage scoring standard table and individual patient's cancer stage, growth rate, AUC, AUC Index, and CODRP Index

**
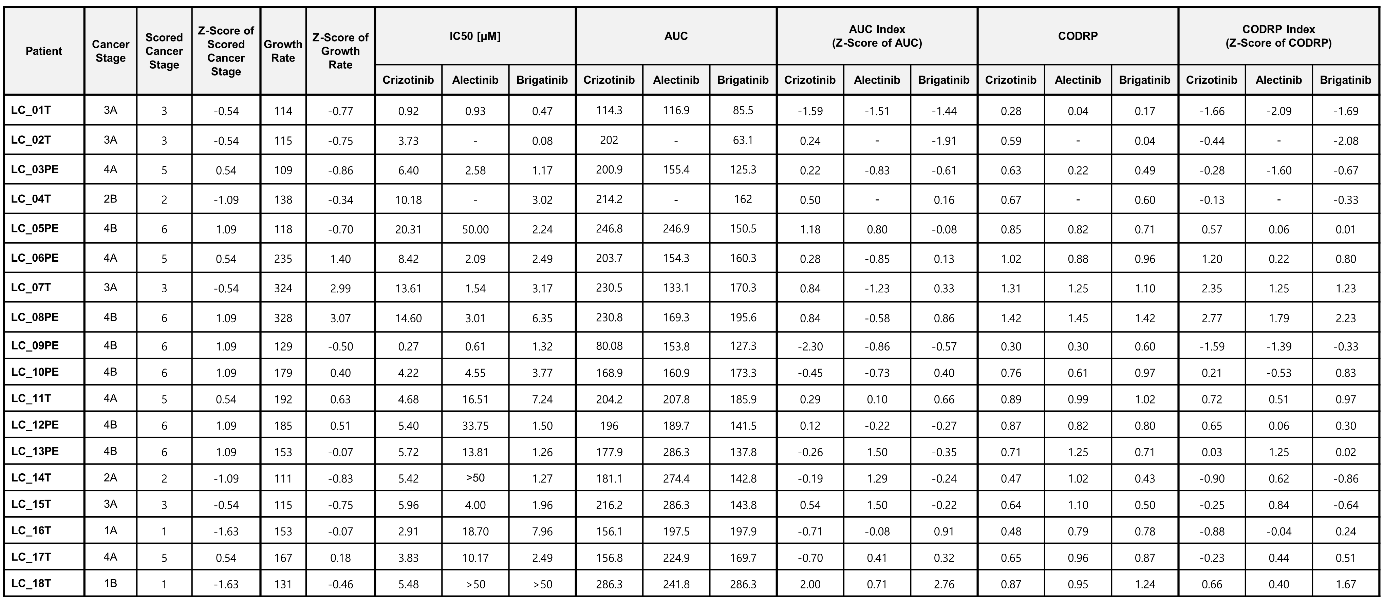
**

**Table S2.** Scoring table of cancer stage for CODRP index

**
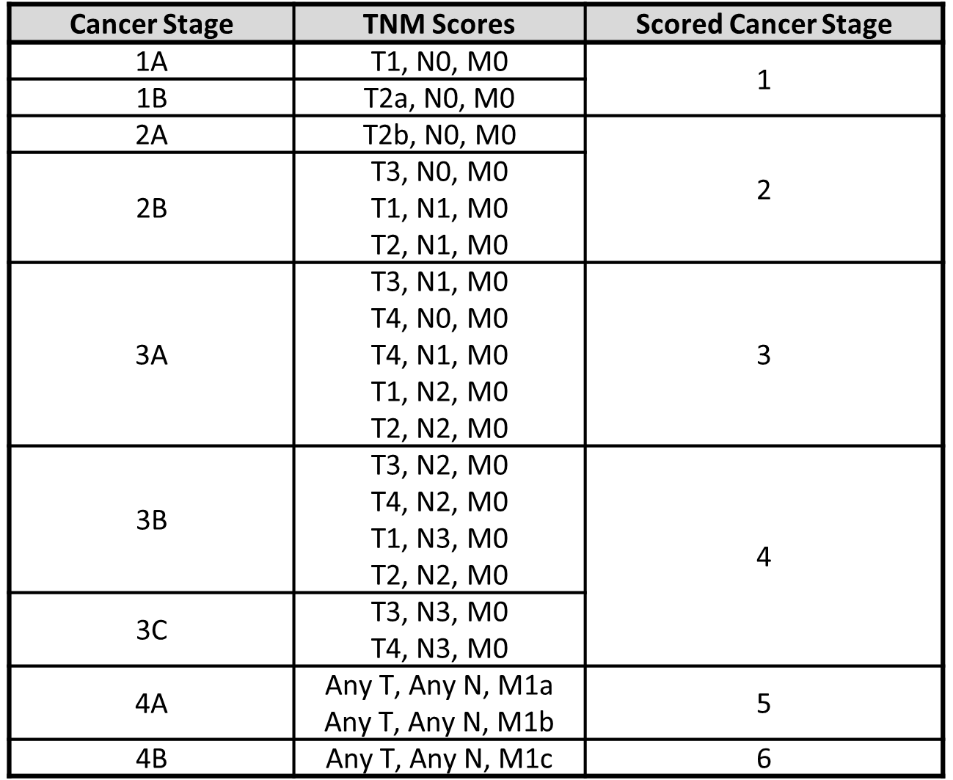
**

**Table S3.** Recipe of the lung cancer PDO culture media

**
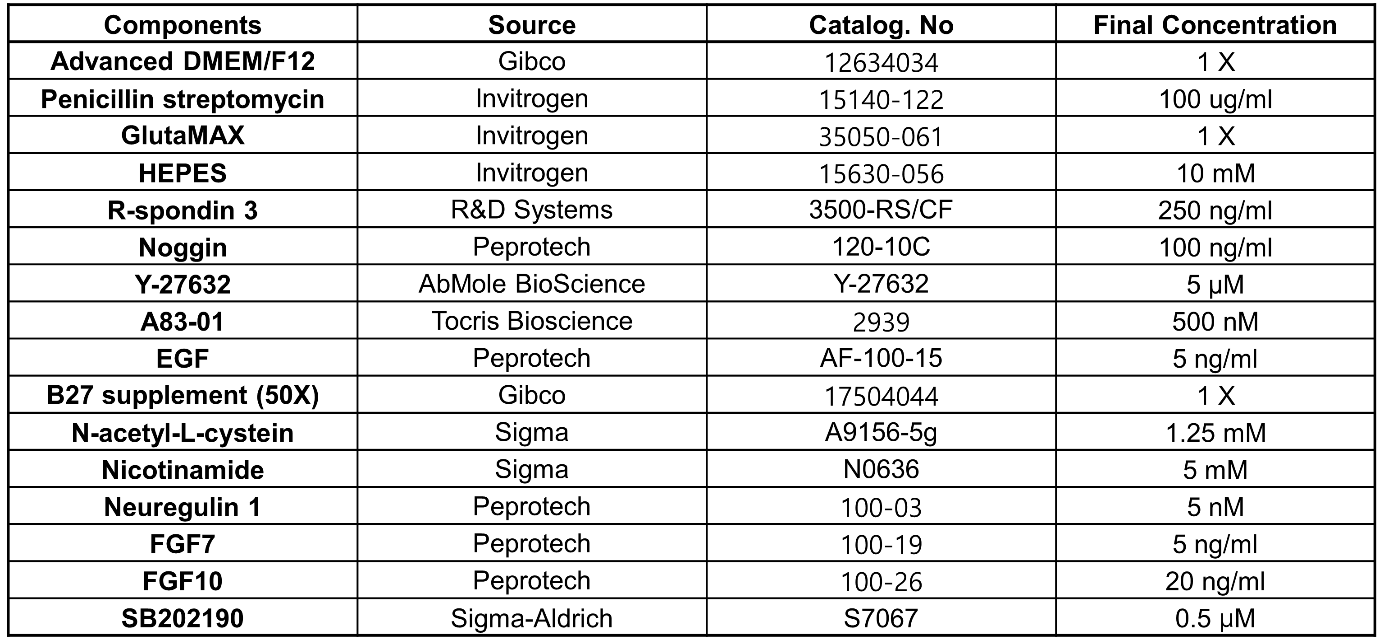
**

**Table S4.** Summary information of lung cancer patient-derived samples and dissociated PDC (viability and total cell number)


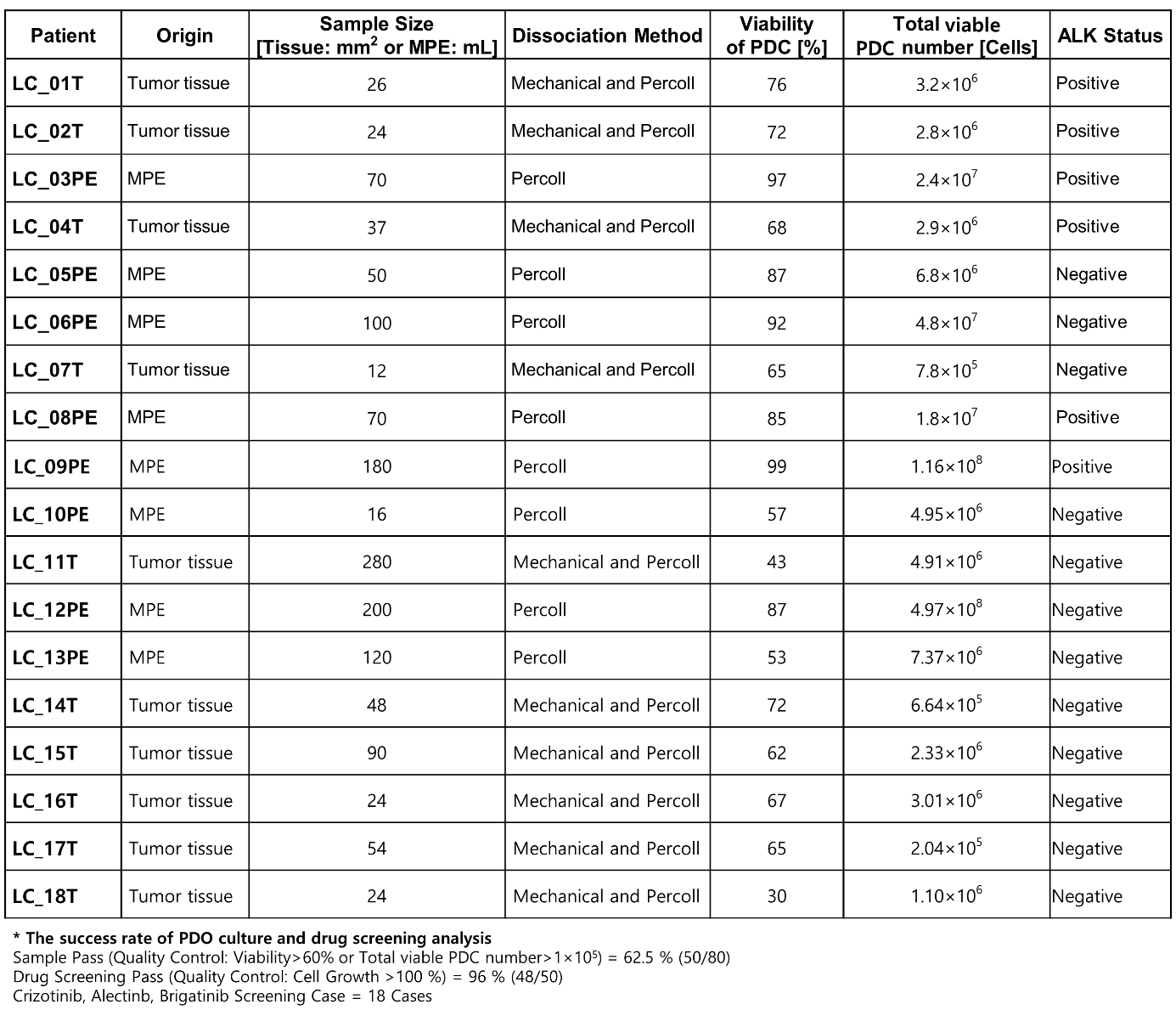


**Table S5.** Summary of pathology analysis

**
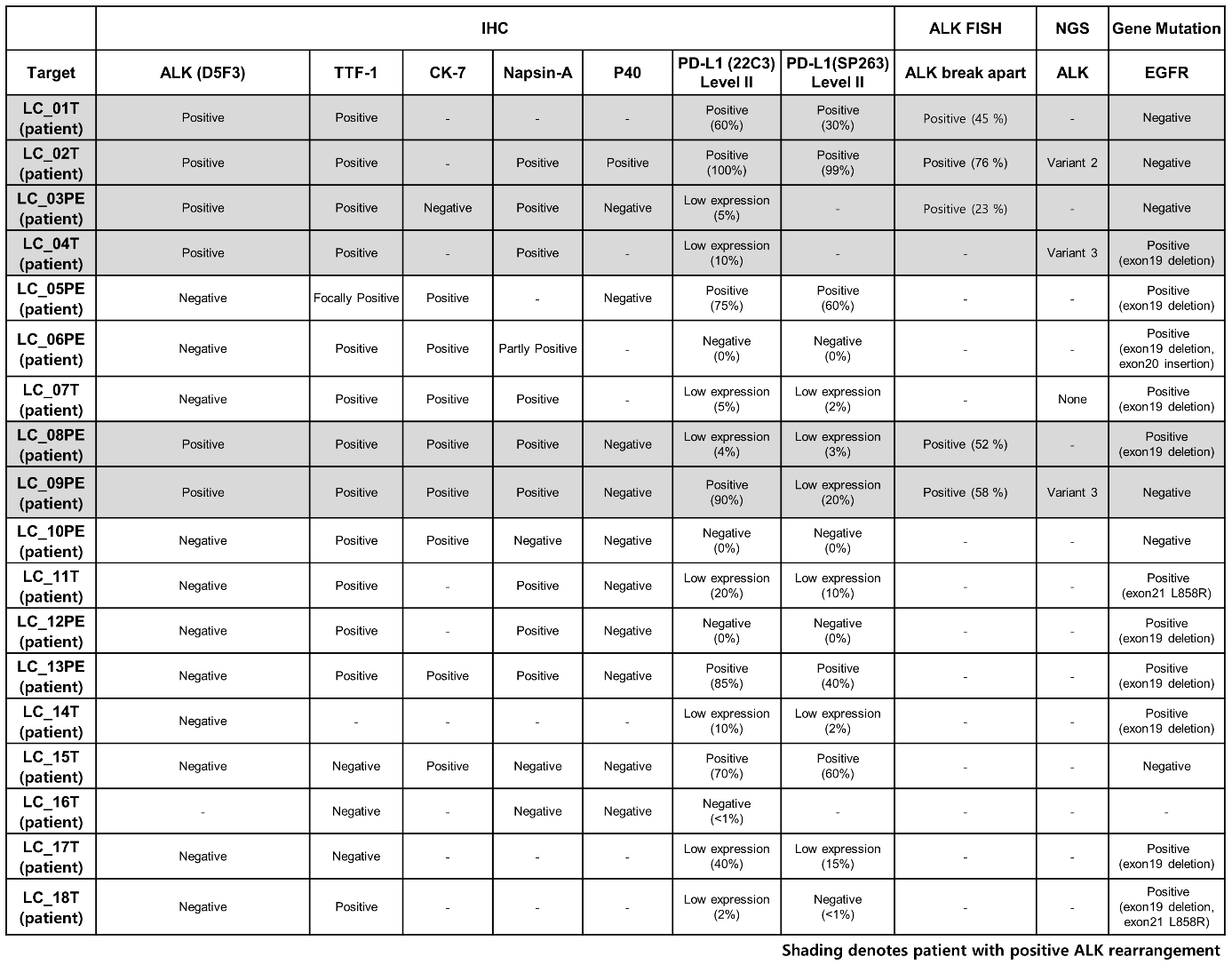
**

**Table S6.** Summary of clinical information


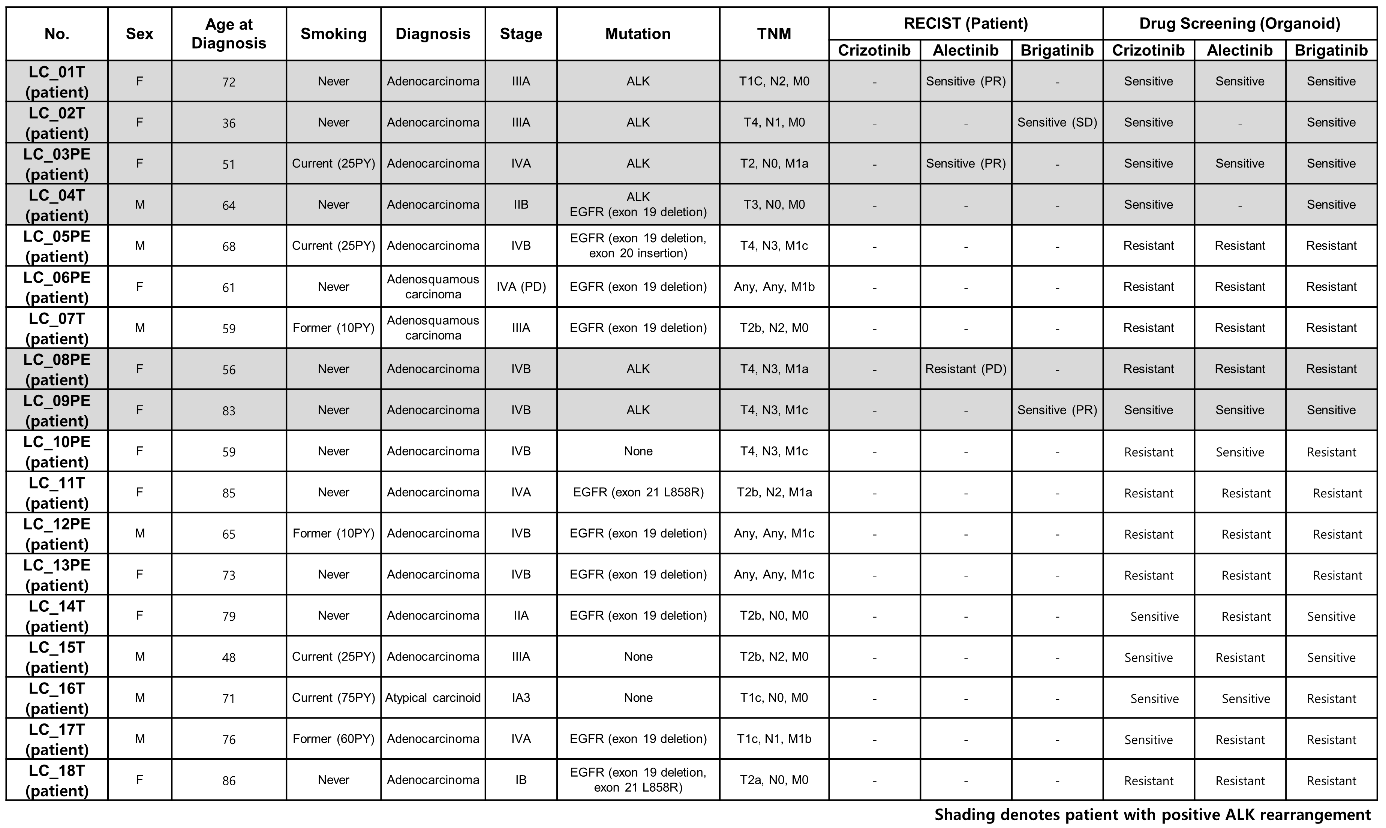


**Table S7.** Summary of multiple linear regression (MLR) analysis results


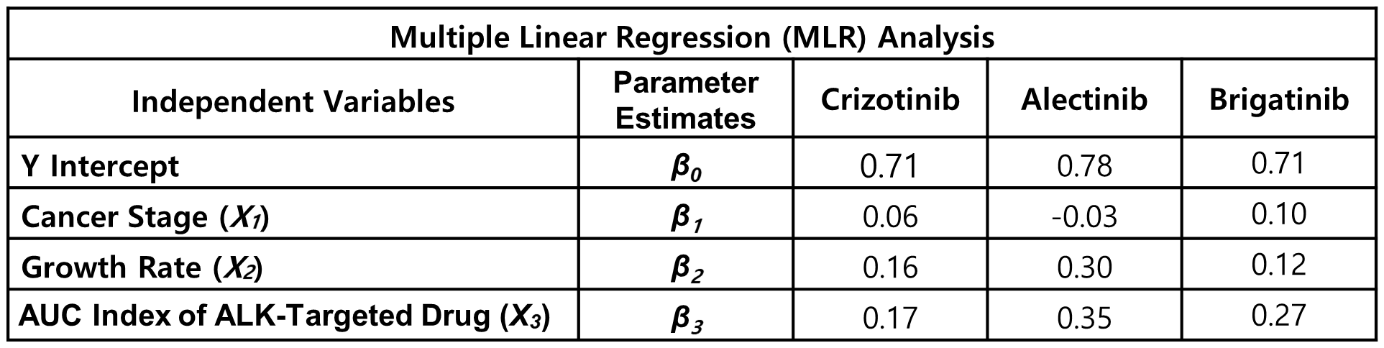

Supplement: Supplementary file 1 — Supplementary Material 1: Figure S1. Summary of Illustration and Limitations for the Conventional 3D-Cell Spotter. (A) A conventional stainless nozzle-type cell spotter that dispenses samples with the pressure of a syringe pump. A dead volume of 50 μL or more is required to dispense high-viscosity samples. In addition, a nozzle-washing process is required to prevent cross-contamination whenever a sample is changed. Figure S2. Comparison of Patient-derived Cell Preparation Methods by Enzymatic and Mechanical Dissociation. (A) Image of organoid culture and proliferation rate difference analysis according to enzymatic and mechanical dissociation methods. (B) Quantitative analysis of differences in lung cancer organoid growth rates according to different dissociation methods through 3D live cell-staining. Figure S3. Results of CODRP index-based drug sensitivity test for each ALK-targeted drug. Drug response to three types of ALK-targeted drugs; (A) crizotinib, (B) alectinib and (C) brigatinib was analyzed into sensitive and resistant areas according to the ALK status of PDOs. Table S1. Cancer stage scoring standard table and individual patient’s cancer stage, growth rate, AUC, AUC Index, and CODRP Index. Table S2. Scoring table of cancer stage for CODRP index. Table S3. Recipe of the lung cancer PDO culture media. Table S4. Summary information of lung cancer patient-derived samples and dissociated PDC (viability and total cell number). Table S5. Summary of pathology analysis. Table S6. Summary of clinical information. Table S7. Summary of multiple linear regression (MLR) analysis results [file 13046_2023_2899_MOESM1_ESM.docx]
